# Herbal Medicine for Behavioral and Psychological Symptoms of Dementia: A Systematic Review and Meta-Analysis

**DOI:** 10.3389/fphar.2021.713287

**Published:** 2021-07-27

**Authors:** Chan-Young Kwon, Boram Lee

**Affiliations:** ^1^ Department of Oriental Neuropsychiatry, Dong-Eui University College of Korean Medicine, Busan, South Korea; ^2^ Department of Clinical Korean Medicine, Graduate School, Kyung Hee University, Seoul, South Korea

**Keywords:** dementia, BPSD, EATM, herbal medicine, systematic review

## Abstract

**Background:** Dementia is a global health concern, causing serious health and socioeconomic burdens with population aging. The associated symptoms of dementia, called behavioral and psychological symptoms of dementia (BPSD), are factors contributing to the socioeconomic burden of dementia. Recently, herbal medicine (HM) has attracted attention as a potential complementary therapy for BPSD. Therefore, this systematic review was aimed at analyzing the effectiveness (or efficacy), safety, and research status of HM in BPSD management through a comprehensive review.

**Methods:** Thirteen electronic databases were searched comprehensively. Related clinical studies published until December 28, 2020, were collected. The methodological quality was evaluated using tools such as the Cochrane Collaboration’s risk of bias tool according to the study design. The effectiveness (or efficacy) was analyzed for randomized controlled trials (RCTs) only, and when sufficient homogeneity was assured, effect estimates were presented as mean difference (MD) and risk ratio (RR), with 95% confidence interval (CIs), through a meta-analysis.

**Results:** A total of 52 clinical studies, including 36 RCTs, were included in this review. As an adjunctive therapy, HM showed statistically significant benefits in BPSD severity assessed by the Behavior Pathology in Alzheimer’s Disease Rating Scale (combined with psychotropic drugs: MD = −3.48, 95% CI: −3.96 to −2.99; with anti-dementia drugs: MD = −2.81, 95% CI: −3.17 to −2.45) and Neuropsychiatric Inventory (with anti-dementia drugs: MD = −3.23, 95% CI: −4.06 to −2.40). Adverse events were significantly less frequent in the HM group (RR = 0.50; 95% CI: 0.28 to 0.88). However, the methodological quality of the RCTs included in this systematic review was not optimal overall.

**Conclusion:** According to the findings of this review, HM may be associated with additional benefits in BPSD treatment, particularly when used as an adjunct to conventional medications, including psychotropic and anti-dementia drugs. However, considering the methodological quality of the included RCTs, this clinical evidence is not robust. Nevertheless, dementia is a global health concern, and considering the limitations of conventional psychotropic drugs for BPSD, a major cause of the disease burden, HM appears to be a promising complementary therapy that warrants further research.

## Introduction

Dementia is a global health concern, causing serious health and socioeconomic burdens with population aging. A study comparing its prevalence and costs between 2010 and 2015 calculated overall annual trends and predicted that the worldwide costs of dementia in 2030 would reach approximately US $2 trillion (Wimo et al., 2017). The clinical manifestation of dementia can be classified into cognitive decline, i.e., core symptoms, and associated symptoms called behavioral and psychological symptoms of dementia (BPSD) (Ohno et al., 2019). BPSD is a term that encompasses various behavioral problems and psychological symptoms that may occur in patients with dementia and is related to the poor patient prognosis, burden of caregivers, and risk of institutionalization, consequently contributing to the socioeconomic burden of dementia (Cerejeira et al., 2012). BPSD is present in most patients with dementia, particularly hyperactivity, apathy, depression, and anxiety, with moderate or higher incidence (van der Linde et al., 2016).

Although pharmacological approaches, including psychotropic drugs, are frequently used to manage BPSD in clinical settings (Ozaki et al., 2017), the results are occasionally unsatisfactory, and drugs such as antipsychotics, benzodiazepines, and Z-drugs are associated with adverse events (AEs), such as increased risk of falls and all-cause mortality (Landi et al., 2005; Ralph and Espinet, 2018). Moreover, patients with dementia are mostly elderly, and the use of several psychotropic drugs in the population is considered a “potentially inappropriate medication,” which discourages the use of psychotropic drugs for BPSD (By the 2019 American Geri, 2019). Therefore, more effective and safe treatments for BPSD management are necessary.

East Asian traditional medicine (EATM) is a medical system that has been established in Asian countries for a long time, and some countries, such as Korea, Japan, China, and Taiwan, use it in their national medical systems (Park et al., 2012). As an EATM modality, herbal medicine (HM) is considered to be a management strategy for dementia, particularly BPSD. For example, an HM called Yokukansan is effective against the positive symptoms of BPSD (Matsuda et al., 2013). However, other types of HMs can also be considered in the management of BPSD, highlighting the need for a comprehensive review of the various HMs that can be used in BPSD (Howes et al., 2017). Therefore, this systematic review was aimed at analyzing the effectiveness (or efficacy), safety, and research status of HM in BPSD management through a comprehensive review.

## Materials and Methods

We registered the protocol of this systematic review in the OSF registries (URL: https://osf.io/3u8ch) and International Prospective Register of Systematic Reviews (URL: https://www.crd.york.ac.uk/prospero/display_record.php?ID=CRD42020211000) before beginning the study. The study protocol was as previously described (Kwon et al., 2021). No amendments were made to the information provided in the protocol. We report the systematic review in accordance with the Preferred Reporting Items for Systematic Review and Meta-Analysis 2020 checklist (Page et al., 2021).

### Information Sources and Search Strategy

One researcher (B Lee) searched MEDLINE *via* PubMed, EMBASE *via* Elsevier, the Cochrane Central Register of Controlled Trials, Allied and Complementary Medicine Database *via* EBSCO, Cumulative Index to Nursing and Allied Health Literature *via* EBSCO, PsycARTICLES *via* ProQuest, Oriental Medicine Advanced Searching Integrated System, Koreanstudies Information Service System, Research Information Service System, Korean Medical Database, Korea Citation Index, China National Knowledge Infrastructure, and Wanfang Data on December 28, 2020. Articles published from the inception of the database to the search date were screened. We also identified additional eligible articles through reviews of relevant literature reference lists and trial registries, such as clinicaltrials.gov, and consultation with experts in this area to include additional gray literature. The detailed search strategies are described in Supplementary Material S1.

### Eligibility Criteria

We included all types of original clinical studies, including randomized controlled clinical trials (RCTs), non-randomized controlled clinical trials (CCTs), and before–after studies without restrictions on the publication language or publication status. Studies involving patients with any type of dementia in long-term care facilities, community, or specialized geriatric assessments and psychiatric units were included. Although there were no restrictions on the sex, age, or race of the participants, studies that did not provide diagnostic criteria or a validated assessment tool for inclusion and studies on patients with drug allergies or other serious illnesses, such as cancer, liver disease, or kidney disease, were excluded. We included studies involving oral HM based on EATM theories as a monotherapy or adjunctive therapies to psychotropic drugs, with or without routine care for dementia as treatment interventions. Although there were no restrictions on the dosage form of HM, we excluded studies that did not list the composition of HM, except for patent drugs. For the control intervention, we included studies involving wait-list, placebo, or psychotropic drugs, with or without routine care for dementia, such as anti-dementia drugs.

The primary outcome was the severity of BPSD symptoms, such as scores of the Behavior Pathology in Alzheimer’s Disease Rating Scale (BEHAVE-AD) (Sclan et al., 1996), Neuropsychiatric Inventory (NPI) (Cummings et al., 1994), and Brief Psychiatric Rating Scale (BPRS) (Overall and Gorham, 1962). The secondary outcomes included 1) total effective rate (TER) for BPSD symptoms; 2) activities of daily living (ADLs) of patients, such as the Barthel Index (Mahoney and Barthel, 1965) and the Functional Independence Measure (Linacre et al., 1994), as well as instrumental ADL (IADL), such as the Activities of Daily Living Prevention Instrument (Galasko et al., 2006); 3) quality of life (QoL) of patients, such as the Alzheimer Disease Related Quality of Life (Kasper et al., 2009); 4) caregiver burden of caregivers, such as the Caregiver Burden Inventory (Novak and Guest, 1989); 5) QoL of caregivers, such as the Short Form 36 Health Survey (Ware and Sherbourne, 1992); 6) placement in a long-term care facility from home; and 7) safety data, such as incidence of AEs.

### Study Selection

All documents retrieved from the databases and other sources were imported into EndNote X8 (Clarivate Analytics, Philadelphia, United States). Using “Find Duplicates” function in EndNote X8 and manual searching, duplicate documents were excluded, and two researchers (CY Kwon and B Lee) independently reviewed the possibility of inclusion by reviewing the titles and abstracts. For the first included documents, the final documents to be included were determined through a review of full texts. Disagreements between the two researchers in the study selection process were resolved through consensus.

### Data Extraction

Two researchers (CY Kwon and B Lee) independently extracted the data from the included studies using a pre-defined form in Excel 2016 (Microsoft, Redmond, WA, United States). The extracted information included the first author’s name, publication year, country, sample size and dropout, details of participants, treatment and control intervention, duration of intervention, main outcome measures and results after treatment ended, AEs, and information to assess the risk of bias (RoB). When the data in each included study were insufficient, we contacted the corresponding authors of the original studies *via* e-mail. Disagreements between the researchers in the data extraction process were resolved through consensus.

### RoB Assessment

To assess the RoB of the included RCTs, we used Cochrane Collaboration’s RoB tool comprising domains of random sequence generation, allocation concealment, blinding of participants, personnel, and outcome assessors, completeness of outcome data, selective reporting, and other biases. In particular, we assessed other bias items based on the statistical baseline imbalance between the treatment and control groups, such as the participant’s mean age, sex, disease period, or disease severity. Each domain was assessed as “low risk,” “unclear risk,” or “high risk” (Higgins, 2011), and the evaluation results are presented as a figure using Review Manager software, version 5.4 (Cochrane, London, United Kingdom). For included CCTs, before–after studies, and case reports, we used the Risk Of Bias In Non-randomized Studies of Interventions tool (Sterne et al., 2016), The Quality Assessment Tool for Before–After (Pre–Post) Studies With No Control Group National Heart, Lung, and Blood Institute (NHLBI) (2013), and the Quality Assessment Tool for Case Series Studies National Heart, Lung, and Blood Institute (NHLBI) (2013), respectively. Two researchers (CY Kwon and B Lee) independently assessed the RoB of the included studies, and discrepancies were resolved through consensus.

### Data Synthesis and Analysis

A descriptive analysis of the findings, including the demographic characteristics of the participants, details of the interventions, and outcomes, were conducted for all included studies. If there were two or more studies using the same type of treatment and control interventions, with the same outcome measures among our primary and secondary outcomes, a meta-analysis was conducted using Review Manager software (version 5.4; Cochrane, London, United Kingdom). For continuous and binary outcomes, the mean difference (MD) and risk ratio (RR) were calculated with 95% confidence interval (CI). We assessed heterogeneity using both the χ^2^ test and the I^2^ statistic, and I^2^ values greater than 50 and 75% were interpreted as substantial and considerable heterogeneity, respectively. We pooled the meta-analyzed results using a random-effects model if the included studies had significant heterogeneity (I^2^ > 50%) and a fixed-effect model if the heterogeneity was insignificant or if less than five studies were included in the meta-analysis because of lack of precision in the estimate of the between-study variance (Guyatt et al., 2002; Balshem et al., 2011). We planned subgroup analyses according to the severity of dementia, type of dementia, severity of BPSD, and treatment duration, if necessary data were available. The Mini-Mental State Examination score was used to classify the severity of dementia of the participants, with scores of 20–24, 13–20, and 12 or less regarded as mild, moderate, and severe, respectively. Additionally, we conducted a sensitivity analysis to identify the robustness of the results of the meta-analysis by excluding 1) studies with high RoB and 2) outliers that are numerically distant from the rest of the data. If more than ten studies were included in each meta-analysis, we planned to assess the publication bias using a funnel plot.

## Results

### Study Selection

**FIGURE 1:**
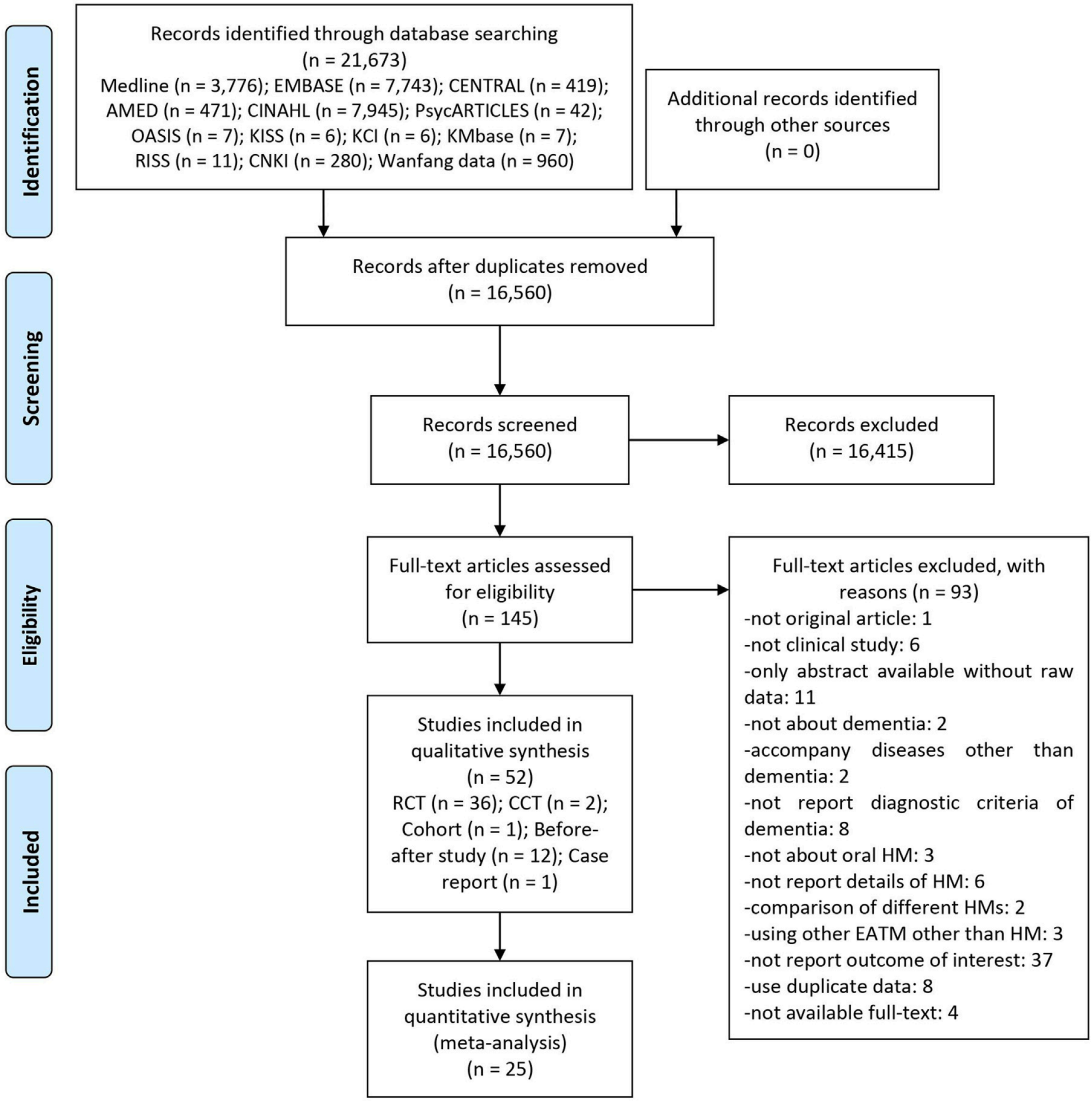
A PRISMA flow diagram of the literature screening and selection processes. AMED, Allied and Complementary Medicine Database; CENTRAL, Cochrane Central Register of Controlled Trials; CCT, non-randomized controlled clinical trial; CINAHL, Cumulative Index to Nursing and Allied Health Literature; CNKI, China National Knowledge Infrastructure; HM, herbal medicine; KCI, Korea Citation Index; KISS, Koreanstudies Information Service System; KMbase, Korean Medical Database; OASIS, Oriental Medicine Advanced Searching Integrated System; RCT, randomized controlled trial; RISS, Research Information Service System; TCM, traditional Chinese medicine.

A total of 21,673 articles were identified through the database search, and there were no additional records from other sources. After removing duplicates, the titles and abstracts of 16,560 articles were screened for inclusion. After excluding 16,415 articles, the full texts of the remaining 145 articles were assessed for final inclusion. We excluded a total of 93 articles, including one for not being an original article, six for not being clinical studies, 11 for being only abstracts without raw data, two for not being about dementia, three for having accompanying diseases other than dementia, eight for not reporting diagnostic criteria of dementia, three for not being about oral HM, six for not reporting details of HM, two for comparing different HMs, three for using traditional Chinese medicine other than HM, 36 for not reporting the outcome of interest, eight for using duplicate data, and four for unavailable full-texts (Supplementary Material S2). Finally, we reviewed 52 studies, including 36 RCTs (Chen et al., 1997; Terasawa et al., 1997; Motohashi, 2006; Mizukami et al., 2009; Monji et al., 2009; Okahara et al., 2010; Guo et al., 2011a; Zhang, 2012; Chen et al., 2013; Shen, 2013; Teranishi et al., 2013; Pan et al., 2014; Pu et al., 2014; Yao et al., 2014; Zhang et al., 2015a; Du et al., 2015; Hu et al., 2015; Liu et al., 2015; Zhou, 2015; Zhou and Wei, 2015; Lin et al., 2016; Furukawa et al., 2017; Zuo, 2017; Fang et al., 2018; Gu et al., 2018; Han, 2018; Li et al., 2018; Shen et al., 2018; Zhang et al., 2018; Zhou, 2018; Huang and Xu, 2019; Shen et al., 2019; Zhu et al., 2019; Chen, 2020; Li, 2020; Shi et al., 2020), two CCTs (Kudoh et al., 2016; Xu, 2018), one cohort (Meguro and Yamaguchi, 2018), 12 before–after studies (Iwasaki et al., 2005; Xu et al., 2007; Shinno et al., 2008; Hayashi et al., 2010; Kawanabe et al., 2010; Guo et al., 2011b; Iwasaki et al., 2012; Nagata et al., 2012; Yang et al., 2012; Sumiyoshi et al., 2013; Ohsawa et al., 2017; Manabe, 2020), and one case report (Shinno et al., 2007). Among them, 25 RCTs (Terasawa et al., 1997; Monji et al., 2009; Guo et al., 2011a; Zhang, 2012; Chen et al., 2013; Shen, 2013; Pan et al., 2014; Pu et al., 2014; Zhang et al., 2015a; Du et al., 2015; Liu et al., 2015; Zhou, 2015; Zhou and Wei, 2015; Lin et al., 2016; Furukawa et al., 2017; Zuo, 2017; Fang et al., 2018; Gu et al., 2018; Han, 2018; Li et al., 2018; Shen et al., 2018; Huang and Xu, 2019; Zhu et al., 2019; Li, 2020; Shi et al., 2020) were included in the meta-analysis (Figure 1).

### Study Characteristics

Thirty-four studies (Chen et al., 1997; Chen et al., 2013; Chen, 2020; Du et al., 2015; Fang et al., 2018; Gu et al., 2018; Guo et al., 2011a; Han, 2018; Hu et al., 2015; Huang and Xu, 2019; Li, 2020; Li et al., 2018; Lin et al., 2016; Liu et al., 2015; Motohashi, 2006; Pan et al., 2014; Pu et al., 2014; Shen, 2013; Shen et al., 2019; Shen et al., 2018; Shi et al., 2020; Yao et al., 2014; Zhang et al., 2015a; Zhang et al., 2018; Zhang, 2012; Zhou, 2018; Zhou, 2015; Zhou and Wei, 2015; Zhu et al., 2019; Zuo, 2017; Xu, 2018; Guo et al., 2011b; Xu et al., 2007; Yang et al., 2012) were published in China, and 18 studies (Furukawa et al., 2017; Mizukami et al., 2009; Monji et al., 2009; Okahara et al., 2010; Teranishi et al., 2013; Terasawa et al., 1997; Kudoh et al., 2016; Meguro and Yamaguchi, 2018; Hayashi et al., 2010; Iwasaki et al., 2012; Iwasaki et al., 2005; Kawanabe et al., 2010; Manabe, 2020; Nagata et al., 2012; Ohsawa et al., 2017; Shinno et al., 2008; Sumiyoshi et al., 2013; Shinno et al., 2007) were published in Japan. The type of dementia was Alzheimer’s disease, vascular dementia, two or more types of dementia, and dementia with Lewy bodies in 26 (Chen, 2020; Fang et al., 2018; Furukawa et al., 2017; Gu et al., 2018; Guo et al., 2011a; Han, 2018; Hu et al., 2015; Li, 2020; Li et al., 2018; Lin et al., 2016; Liu et al., 2015; Monji et al., 2009; Okahara et al., 2010; Pan et al., 2014; Zhang et al., 2015a; Zhang et al., 2018; Zhou, 2018; Zhou, 2015; Zhou and Wei, 2015; Zuo, 2017; Kudoh et al., 2016; Meguro and Yamaguchi, 2018; Guo et al., 2011b; Hayashi et al., 2010; Ohsawa et al., 2017; Yang et al., 2012), 12 (Chen et al., 1997; Motohashi, 2006; Pu et al., 2014; Shen et al., 2019; Shen et al., 2018; Shi et al., 2020; Terasawa et al., 1997; Yao et al., 2014; Zhu et al., 2019; Xu, 2018; Nagata et al., 2012; Xu et al., 2007), five (Chen et al., 2013; Du et al., 2015; Shen, 2013; Zhang, 2012; Sumiyoshi et al., 2013), and four (Iwasaki et al., 2012; Iwasaki et al., 2005; Manabe, 2020; Shinno et al., 2007) studies, respectively. Among RCTs, HM was evaluated as a monotherapy, control, psychotropic drug, and placebo in 16 (Chen et al., 1997; Chen et al., 2013; Furukawa et al., 2017; Mizukami et al., 2009; Pan et al., 2014; Pu et al., 2014; Shen, 2013; Shi et al., 2020; Teranishi et al., 2013; Terasawa et al., 1997; Zhang et al., 2015a; Zhang, 2012; Zhou, 2018; Zhou, 2015; Zhou and Wei, 2015; Zuo, 2017), nine (Chen et al., 2013; Pu et al., 2014; Shen, 2013; Teranishi et al., 2013; Zhang et al., 2015a; Zhang, 2012; Zhou, 2018; Zhou, 2015; Zuo, 2017), and four (Furukawa et al., 2017; Pan et al., 2014; Shi et al., 2020; Terasawa et al., 1997) studies, respectively. Twenty studies (Chen, 2020; Du et al., 2015; Fang et al., 2018; Gu et al., 2018; Guo et al., 2011a; Han, 2018; Hu et al., 2015; Huang and Xu, 2019; Li, 2020; Li et al., 2018; Lin et al., 2016; Liu et al., 2015; Monji et al., 2009; Motohashi, 2006; Okahara et al., 2010; Shen et al., 2019; Shen et al., 2018; Yao et al., 2014; Zhang et al., 2018; Zhu et al., 2019) evaluated HM as an adjunctive therapy. Anti-dementia drugs were the most used as a control group in 11 studies (Chen, 2020; Du et al., 2015; Fang et al., 2018; Guo et al., 2011a; Han, 2018; Hu et al., 2015; Li, 2020; Liu et al., 2015; Okahara et al., 2010; Shen et al., 2018; Zhu et al., 2019), followed by psychotropic drugs in six studies (Gu et al., 2018; Huang and Xu, 2019; Li et al., 2018; Lin et al., 2016; Monji et al., 2009; Zhang et al., 2018). In a total of 13 studies (Chen et al., 2013; Guo et al., 2011a; Han, 2018; Hu et al., 2015; Huang and Xu, 2019; Lin et al., 2016; Pu et al., 2014; Yao et al., 2014; Zhang et al., 2018; Zhou, 2015; Zhu et al., 2019; Guo et al., 2011b; Yang et al., 2012), participants were recruited according to pattern identification, of which blood stasis was the most common (six studies) (Chen et al., 2013; Hu et al., 2015; Pu et al., 2014; Yao et al., 2014; Zhu et al., 2019; Yang et al., 2012), followed by phlegm (five studies) (Chen et al., 2013; Hu et al., 2015; Lin et al., 2016; Zhu et al., 2019; Yang et al., 2012) or kidney deficiency (five studies) (Chen et al., 2013; Guo et al., 2011a; Hu et al., 2015; Huang and Xu, 2019; Guo et al., 2011b). The treatment period ranged from 2 weeks to 2 years, of which 4 weeks (1 month) was the most common in 20 studies (Chen et al., 2013; Chen, 2020; Furukawa et al., 2017; Guo et al., 2011a; Li, 2020; Mizukami et al., 2009; Okahara et al., 2010; Zhou, 2018; Xu, 2018; Guo et al., 2011b; Hayashi et al., 2010; Iwasaki et al., 2012; Iwasaki et al., 2005; Kawanabe et al., 2010; Manabe, 2020; Nagata et al., 2012; Shinno et al., 2008; Sumiyoshi et al., 2013; Yang et al., 2012; Shinno et al., 2007), followed by 8 weeks (2 months) in 12 studies (Chen et al., 1997; Fang et al., 2018; Huang and Xu, 2019; Lin et al., 2016; Motohashi, 2006; Pu et al., 2014; Shen et al., 2019; Shen et al., 2018; Teranishi et al., 2013; Zhang, 2012; Zhu et al., 2019; Zuo, 2017) and 12 weeks (3 months) in 10 studies (Du et al., 2015; Gu et al., 2018; Han, 2018; Li et al., 2018; Monji et al., 2009; Shen, 2013; Terasawa et al., 1997; Yao et al., 2014; Zhou and Wei, 2015; Ohsawa et al., 2017). After completion of treatment, the follow-up was performed in four studies (Fang et al., 2018; Pan et al., 2014; Zhang et al., 2015a; Kawanabe et al., 2010), of which the duration was 4 weeks in two studies (Fang et al., 2018; Kawanabe et al., 2010) and 5 (Pan et al., 2014) and 24 weeks (Zhang et al., 2015a) in one study each. Eighteen studies (Furukawa et al., 2017; Lin et al., 2016; Mizukami et al., 2009; Monji et al., 2009; Okahara et al., 2010; Pan et al., 2014; Shi et al., 2020; Teranishi et al., 2013; Zhang et al., 2015a; Kudoh et al., 2016; Meguro and Yamaguchi, 2018; Hayashi et al., 2010; Iwasaki et al., 2012; Manabe, 2020; Nagata et al., 2012; Ohsawa et al., 2017; Shinno et al., 2008; Sumiyoshi et al., 2013) were approved by the institutional review board before the study began, and 37 studies (Chen, 2020; Du et al., 2015; Furukawa et al., 2017; Gu et al., 2018; Han, 2018; Hu et al., 2015; Huang and Xu, 2019; Li, 2020; Lin et al., 2016; Liu et al., 2015; Mizukami et al., 2009; Monji et al., 2009; Okahara et al., 2010; Pan et al., 2014; Pu et al., 2014; Shen, 2013; Shen et al., 2019; Shen et al., 2018; Shi et al., 2020; Teranishi et al., 2013; Terasawa et al., 1997; Zhang et al., 2015a; Zhang et al., 2018; Zhou, 2018; Zhou, 2015; Zhu et al., 2019; Zuo, 2017; Kudoh et al., 2016; Meguro and Yamaguchi, 2018; Hayashi et al., 2010; Iwasaki et al., 2012; Iwasaki et al., 2005; Manabe, 2020; Nagata et al., 2012; Ohsawa et al., 2017; Shinno et al., 2008; Sumiyoshi et al., 2013) received consent forms from participants (Table 1, Supplementary Material S3). Various types of HMs were used in the included studies, of which Yokukansan was the most frequently used in 13 studies (Iwasaki et al., 2005; Shinno et al., 2007; Shinno et al., 2008; Mizukami et al., 2009; Monji et al., 2009; Hayashi et al., 2010; Kawanabe et al., 2010; Okahara et al., 2010; Iwasaki et al., 2012; Nagata et al., 2012; Sumiyoshi et al., 2013; Teranishi et al., 2013; Furukawa et al., 2017), followed by Xiaoyaosan (four studies) (Zhou, 2015; Zhou and Wei, 2015; Shen et al., 2018; Shen et al., 2019) and Liuweidihuang pill (three studies (Shen, 2013; Du et al., 2015; Gu et al., 2018). In terms of dosage form, powder was most often used in 18 studies (Terasawa et al., 1997; Iwasaki et al., 2005; Shinno et al., 2007; Mizukami et al., 2009; Monji et al., 2009; Hayashi et al., 2010; Kawanabe et al., 2010; Okahara et al., 2010; Iwasaki et al., 2012; Nagata et al., 2012; Sumiyoshi et al., 2013; Teranishi et al., 2013; Zhou and Wei, 2015; Kudoh et al., 2016; Furukawa et al., 2017; Ohsawa et al., 2017; Meguro and Yamaguchi, 2018; Manabe, 2020), followed by decoction (16 studies) (Guo et al., 2011a; Guo et al., 2011b; Yang et al., 2012; Zhang, 2012; Pu et al., 2014; Yao et al., 2014; Zhang et al., 2015a; Hu et al., 2015; Zhou, 2015; Zuo, 2017; Han, 2018; Li et al., 2018; Zhou, 2018; Huang and Xu, 2019; Zhu et al., 2019; Li, 2020), granules (seven studies) (Motohashi, 2006; Xu et al., 2007; Chen et al., 2013; Liu et al., 2015; Xu, 2018; Chen, 2020; Shi et al., 2020), pill (five studies) (Shen, 2013; Du et al., 2015; Gu et al., 2018; Shen et al., 2018; Shen et al., 2019), and capsules (four studies) (Chen et al., 1997; Lin et al., 2016; Fang et al., 2018; Zhang et al., 2018) (Supplementary Material S4).

**TABLE 1:** Basic characteristics of the randomized controlled trial.

Study ID	Sample size (included →analyzed)	Mean age (year)	Sex (M:F)	Population (Diagnosis)	Pattern identification	(A) Treatment intervention	(B) Control intervention	Treatment duration/Follow-up	Outcome
Chen et al. (1997)	61 (32:29)	(A) 64.32 ± 5.42	(A) 32(27:5)	-VD (DSM-IV, ICD-10)	NA	HM	Hydergine 6 mg/day	2 months/NR	1. MMSE
				-MMSE≤23					2. HAMD
	→61(32:29)	(B) 63.21 ± 6.41	(B) 29(21:8)	(A) 19.0 ± 3.4					3. TER (TCM symptom score)
				(B) 18.6 ± 4.5					4. TER (Neurological deficit)
				-HIS<24					5. TER (ADL)
				-HAMD					6. TER (MMSE)
				(A) 18.0 ± 6.4					7. Gait and balance function
				(B) 17.5 ± 6.3					8. Cerebral blood flow
									9. TER (Electroencephalography)
Chen et al. (2013)	60(30:30)	(A) 73.3 ± 5.1	(A) 30(20:10)	-AD or VD (CCMD-3, ICD-10)	Sea of marrow deficiency, dual deficiency of spleen-kidney, liver-kidney deficiency, phlegm turbidity	HM	Risperidone 0.5 mg/day (modification up to 3 mg/day, according to patient’s condition)	4 weeks/NR	1. TER (BEHAVE-AD) 2. BEHAVE-AD
	→60(30:30)	(B) 74.3 ± 7.4	(B) 30(23:7)	-MMSE≤24	obstructing the orifices, blood stasis due to qi stagnation				
				-BEHAVE-AD≥8					
				(A) 16.3 ± 7.3					
				(B) 15.8 ± 6.9					
Chen (2020)	80(40:40)	(A) 74.52 ± 2.65	(A) 40(22:18)	-AD	NA	HM + (B)	Health education, donepezil 5 mg/day	1 month/NR	1. PSQI
	→80(40:40)	(B) 73.82 ± 2.88	(B) 40(24:16)	-Insomnia (DSM-5)					
				-PSQI>7					
Du et al. (2015)	105(51:54)	(A) 74.9	(A) 51(31:20)	-AD or VD (DSM-IV)	NA	HM + (B)	Memantine 5 mg/day	3 months/NR	1. TER (BEHAVE-AD)
				-BEHAVE-AD≥8			(1 week: 5 mg/day, 2 weeks: 10 mg/day, 3 weeks: 15 mg/day, 4 weeks: 20 mg/day)		2. BEHAVE-AD
	→105(51:54)	(B) 74.7	(B) 54(32:22)	(A) 18.3 ± 3.9					3. ACE-R
				(B) 18.7 ± 4.0					4. Barthel index
				-GDS<5					
Fang et al. (2018)	90(45:45)	(A) 71.5 ± 9.1	(A) 45(26:19)	-AD+depression (DSM-IV)	NA	HM + (B)	Donepezil 5 mg/day	8 weeks/4 weeks	1. MoCA
	→90(45:45)	(B) 70.4 ± 8.4	(B) 45(25:20)	-MoCA			(10 mg/day after 4 weeks)		2. HAMD
				(A) 14.44 ± 3.14					3. Serum 5-HT
				(B) 14.82 ± 3.25					4. Serum dopamine
				-HAMD					
				(A) 22.53 ± 3.15					
				(B) 23.11 ± 3.25					
Furukawa et al. (2017)	145(75:70)	(A) 78.3 ± 5.4	(A) 75(33:42)	-AD (NINCDS-ADRDA)	NA	HM	Placebo	4 weeks/NR	1. NPI-Q
	→129(65:64)	(B) 78.5 ± 5.1	(B) 70(28:42)	-NPI-Q>4, agitation/aggression + irritability/lability>2					2. MMSE
				(A) 9.6 ± 4.2					
				(B) 9.4 ± 4.4					
				-MMSE 10-26					
				(A) 19.7 ± 3.9					
				(B) 19.0 ± 4.4					
Gu et al. (2018)	80(40:40)	(A) 66.8 ± 7.6	(A) 40(21:19)	-AD (CCMD)	NA	HM + (B)	Olanzapine 2.5 mg/day	3 months/NR	1. TER (BPRS symptom)
	→80(40:40)	(B) 71.7 ± 6.4	(B) 40(18:22)	-BPRS≥35					2. BPRS
				(A) 63 ± 3			(modification according to patient’s condition)		
				(B) 61 ± 7					
Guo et al. (2011a)	60(30:30)	(A) 73.8 ± 1.02	(A) 30(16:14)	-AD (NINCDS-ADRDA)	Liver-kidney deficiency	HM + (B)	Donepezil 5 mg/day	1 month/NR	1. BEHAVE-AD
	→60(30:30)	(B) 73.14 ± 0.96	(B) 30(18:12)	-BEHAVE-AD≥8					2. TER (BEHAVE-AD)
				(A) 13.77 ± 2.66			(modification according to patient’s condition)		3. MMSE
				(B) 13.57 ± 2.77					
Han (2018)	47 (29:28)	(A) 61.35 ± 6.28	(A) 29(18:11)	-AD (CCMD-3)	qi blood deficiency	HM + (B)	Rivastigmine 3 mg/day	3 months/NR	1. MMSE
	→47(29:28)	(B) 62.38 ± 6.15	(B) 28(16:12)	-MMSE 12-24					2. ADAS-cog
				(A) 18.5 ± 2.7					3. Bathel index
				(B) 18.5 ± 2.8					4. NPI
				-ADAS-cog					5. SDSD
				(A) 11.6 ± 2.6					6. TER (TCM symptom score)
				(B) 12.5 ± 2.4					
Hu et al. (2015)	80(40:40)	(A) 68.4 ± 7.2	(A) 40(26:14)	-AD (NINCDS-ADRDA, DSM-IV)	Kidney essence deficiency, phlegm and stasis obstruction	HM + (B)	Donepezil 10 mg/day, Piracetam 2.4g/day	6 months/NR	1. TER (clinical symptom)
				-MMSE 21-26					2. MMSE
	→80(40:40)	(B) 69.2 ± 6.4	(B) 40(25:15)	(A) 15.28 ± 2.74					3. ADAS-cog
				(B) 15.49 ± 2.87					4. ADL
				-ADAS-cog					5. NPI-Q
				(A) 65.71 ± 7.95					6. Serum SOD
				(B) 64.27 ± 7.36					7. Serum MDA
									8. Serum TNF-α
									9. Serum IL-1
									10. Serum IL-6
Huang and Xu (2019)	90(45:45)	(A) 69.6 ± 5.1	(A) 45(23:22)	-SD (ICD, DSM)	Liver-kidney deficiency, dual deficiency of spleen-kidney, and sea of marrow deficiency	HM + (B)	Olanzapine 2.5 mg/day (modification up to 20 mg/day, according to patient’s condition)	2 months/NR	1. BEHAVE-AD
				-MMSE<17					2. HDS
				(A) 13.3 ± 3.2					3. MMSE
	→90(45:45)	(B) 67.7 ± 4.7	(B) 45(24:21)	(B) 13.8 ± 3.1					4. ADL
				-HDS<16					5. Serum SOD
				(A) 12.7 ± 3.2					6. Serum MDA
				(B) 12.6 ± 3.1					7. Serum IL-6
				-BEHAVE-AD>8					8. Serum IL-1
				-ADL≥22					9. TER (BEHAVE-AD)
Li et al. (2018)	100(50:50)	(A) 69.87 ± 2.65	(A) 50(24:26)	-AD (Textbook in psychiatry for Asia)	NA	HM + (B)	Clonapine 25 mg/day	12 weeks/NR	1. Serum SOD
	→100(50:50)	(B) 68.19 ± 2.73	(B) 50(23:27)	-Behavior disorder			(after 2–3 weeks, +20–50 mg/3–4 days up to 200 mg/day)		2. Serum MDA
									3. BEHAVE-AD
									4. TER (BEHAVE-AD)
Li (2020)	82(41:41)	(A) 69.35 ± 4.08	(A) 41(23:18)	-AD [2018 Guidelines for the diagnosis and treatment of dementia and cognitive impairment in China(2)]	NA	HM + (B)	Donepezil 5 mg/day	1 month/NR	1. TER (Positive and Negative Syndrome Scale, MMSE)
	→82(41:41)	(B) 70.67 ± 3.78	(B) 41(25:16)	-MMSE 10-25					2. MMSE
				(A) 17.32 ± 2.58					3. BEHAVE-AD
				(B) 16.89 ± 2.58					4. Plasma Hcy
									5. Plasma CRP
Lin et al. (2016)	92(46:46)	(A) 71.8 ± 6.4	(A) 46(24:22)	-AD (CCMD-3)	non-interaction between the heart and kidney and phlegm turbidity	HM + (B)	Aripiprazole 2.5 mg/day	8 weeks/NR	1. MMSE
	→84(41:43)	(B) 70.5 ± 6.7	(B) 46(26:20)	-MMSE≤24					2. BEHAVE-AD
				(A) 15.18 ± 3.05					3. TER (BPRS)
				(B) 15.04 ± 3.27					
				-BEHAVE-AD≥8					
Liu et al. (2015)	86(43:43)	(A) 71 ± 4	(A) 43(18:25)	-AD (Guidelines for the diagnosis and treatment of dementia and cognitive impairment in China)	NA	HM + (B)	Donepezil 5 mg/day (modification up to 10 mg/day)	6 months/NR	1. BEHAVE-AD
	→86(43:43)	(B) 72 ± 3	(B) 43(20:23)	-MMSE 10-24					2. CDR
				(A) 17.9 ± 3.0					3. NPI
				(B) 16.9 ± 3.0					4. Plasma 8-isoprostane F2α
				-CDR					5. Urine 8-isoprostane F2α
				(A) 2.00 ± 0.30					
				(B) 1.90 ± 0.20					
Mizukami et al. (2009)	103(53:50)	(A) outpatient 80.6 ± 3.9; inpatient 78.9 ± 6.9	(A) 53(25:5)(B) 50(20:6)	-AD (NINCDS-ADRDA, DSM-IV) or DLB (Consensus guidelines for the clinical and pathologic diagnosis of DLB)	NA	HM	No treatment	4 weeks/NR	1. NPI
				-MMSE					2. MMSE
	→103(53:50)	(B) outpatient 76.9 ± 6.1; inpatient 78.0 ± 6.7		(A) outpatient 17.4 ± 6.3; inpatient 9.8 ± 6.9					3. Barthel index
				(B) outpatient 14.9 ± 5.6; inpatient 9.4 ± 6.7					4. IADL
				-NPI≥6 for at least one of ten items					
				(A) outpatient 25.5 ± 12.0; inpatient 22.1 ± 13.2					
				(B) outpatient 28.6 ± 13.3; inpatient 26.4 ± 16.3					
Monji et al. (2009)	15(10:5)	(A) 80.8 ± 4.7	(A) 10(2:8)	-AD (NINCDS-ADRDA, DSM-IV)	NA	HM + (B)	Sulpiride 50 mg/day (modification according to patient’s condition)	12 weeks/NR	1. NPI
	→14(10:4)	(B) 79.0 ± 2.0	(B) 5(0:5)	-MMSE 6-23					2. dose of sulpiride
				(A) 15.1 ± 4.0					3. MMSE
				(B) 16.4 ± 3.5					4. Barthel index
				-NPI≥6 on at least 1 of the delusions, hallucinations, agitation/aggression, disinhibition, irritability/lability or aberrant motor activity subscales after the treatment with Sulpiride 50 mg/day for 2 weeks					
				(A) 26.7 ± 15.7					
				(B) 22.4 ± 12.8					
Motohashi (2006)	52(28:24)	(A) 80.00 ± 10.66	(A) 28(9:19)	-VD (DSM-IV)	NA	HM + (B)	Routine care (treating primary disease and symptomatic treatment)	8 weeks/NR	1. HDS-R
									2. DAD
									3. BEHAVE-AD
									4. GBS
									5. TER (cognitive symptom)
	→49(26:23)	(B) 85.04 ± 9.64	(B) 24(7:17)	-HDS-R≤20					6. TCM symptom score
									7. Serum lipoprotein(a)
									8. Serum adrenalin
									9. Serum noradrenalin
									10. Serum dopamine
Okahara et al. (2010)	63(30:33)	(A) 76.1 ± 8.1	(A) 29(10:19)	-AD (NINCDS-ADRDA, DSM-IV, ICD-10)	NA	HM + (B)	Donepezil	4 weeks/NR	1. NPI
				-MMSE					
	→61(29:32)	(B) 77.1 ± 6.8	(B) 32(15:17)	(A) 18.3 ± 5.2			(fixed dose during the study)		2. MMSE
				(B) 17.9 ± 5.5					3. DAD
				-NPI (at least one symptom score of four or more in the NPI subscales)					4. Zarit burden interview
				(A) 22.3 ± 10.4					5. SDS
				(B) 21.9 ± 13.9					6. Serum potassium
				-SDS					
				(A) 40.9 ± 7.7					
				(B) 43.7 ± 8.2					
				-HIS≤6					
Pan et al. (2014)	98(49:49)	(A) 57.2 ± 9.7	(A) 45(28:17)	-AD (DSM-IV-TR)	NA	HM	Placebo	20 weeks/5 weeks	1. MMSE
	→91(45:46)	(B) 56.9 ± 10.2	(B) 46(27:19)	-MMSE 10-24					2. BEHAVE-AD
				(A) 13.4 ± 1.8					3. NPI
				(B) 14.1 ± 1.5					4. Actigraphy
Pu et al. (2014)	70(35:35)	(A) 68.79 ± 7.99	(A) 35(20:15)	-VD (CCMD-3)	internal obstruction of static blood	HM	Oxcarbazepine 300–600 mg/day	8 weeks/NR	1. TER (BEHAVE-AD)
	→70(35:35)	(B) 71.34 ± 8.25	(B) 35(22:13)	-MMSE≤26					2. BEHAVE-AD
				(A) 11.36 ± 4.65					3. MMSE
				(B) 11.84 ± 4.52					4. ADL
				-BEHAVE-AD≥8					
				(A) 17.80 ± 6.33					
				(B) 17.49 ± 6.58					
				-HIS>7					
Shen (2013)	110(55:55)	(A) 75.3	(A) 55(32:23)	-AD or VD (CCMD-3)	NA	HM	Olanzapine 2.5 mg/day (modification up to 5–20 mg/day, according to patient’s condition)	3 months/NR	1. TER (BEHAVE-AD)
	→110(55:55)	(B) 74.6	(B) 55(34:21)	-MMSE					2. BEHAVE-AD
				(A) 15.4 ± 3.6					3. MMSE
				(B) 15.7 ± 4.1					
				-BEHAVE-AD≥8					
				(A) 18.4 ± 4.0					
				(B) 18.4 ± 3.69					
				-GDS<5					
Shen et al. (2018)	90(45:45)	(A) 64.36 ± 5.71	(A) 45(25:20)	-VD (2002 Criteria for Vascular Dementia of Neurology Branch of Chinese Medical Association)	NA	HM + (B)	Donepezil 5 mg/day	8 weeks/NR	1. TER (HAMD)
	→90(45:45)	(B) 65.13 ± 6.14	(B) 45(26:19)	-Depression (CCMD-3)					2. MMSE
				-MMSE					3. ADL
				(A) 10.56 ± 2.19					4. HAMD
				(B) 10.78 ± 2.24					5. Serum dopamine
				-HAMD					6. Serum BDNF
				(A) 24.18 ± 2.67					7. Serum Hcy
				(B) 24.35 ± 3.08					
Shen et al. (2019)	100(50:50)	(A) 69.2 ± 11.5	(A) 50(33:17)	-VD (Criteria for Vascular Dementia of Neurology Branch of Chinese Medical Association)	NA	HM + (B)	Oxiracetam 10 mg/day (modification up to 20 mg/day, according to patient’s response.)	8 weeks/NR	1. TER (HAMD)
	→100(50:50)	(B) 67.6 ± 10.6	(B) 50(35:15)	-Depression (CCMD-3)					2. CSDD
				-MMSE					3. CDR
				(A) 17.69 ± 7.91					4. MMSE
				(B) 17.39 ± 6.28					5. ADL
				-CSDD					6. Serum BDNF
				(A) 22.31 ± 4.22					7. Serum S100B
				(B) 21.86 ± 5.65					8. Serum norepinephrine
				-CDR (mild to moderate dementia)					9. Serum dopamine
									10. Serum 5-HT
									11. Serum Hcy
Shi et al. (2020)	543(242:241:60)	(A) 64.72 ± 9.18	(A) 232(154:78)	-VD (NINDS-AIREN)	NA	HM + Donepezil placebo	(B1) HM placebo + Donepezil (B2) HM placebo + Donepezil placebo	24 weeks/NR	1. changes of VADAS-cog
				-MMSE 14-26					2. improvement rate of CIBIC-plus
				(A) 20.56 ± 3.36					3. changes of NPI
				(B1) 20.56 ± 3.24					4. changes of MMSE
	→520(232:233:55)	(B1) 64.31 ± 9.99	(B1) 233(149:84)	(B2) 20.51 ± 2.97					5. changes of TMT-A
				-NPI					6. changes of TMT-B
		(B2) 63.95 ± 9.15	(B2) 55(35:20)	(A) 5.31 ± 5.52					7. changes of ADL
				(B1) 5.35 ± 4.91					8. changes of CDT
				(B2) 5.40 ± 5.51					
				-HIS>7					
Teranishi et al. (2013)	82(27:27:28)	(A) 83.50 ± 5.83	(A) 26(7:19)	-AD or VD or DLB (DSM-IV, NINCDS-ADRDA)	NA	HM	(B1) Risperidone 0.5–2.0 mg/day (modification according to patient’s condition)	8 weeks/NR	1. NPI-NH
	→76(26:25:25)	(B1) 80.72 ± 8.78	(B1) 25(9:16)	-MMSE<19		(Zopiclone (7.5–10 mg/day) and brotizolam (0.25 mg/day), if needed for insomnia)			2. MMSE
				(A) 4.42 ± 4.58					3. FIM
		(B2) 83.20 ± 5.39	(B2) 25(9:16)	(B1) 5.16 ± 5.73			(B2) Fluvoxamine 25–200 mg/day (modification according to patient’s condition)		4. DIEPSS
				(B2) 4.48 ± 5.25					
				-NPI-NH (at least 1 symptom score of greater than 4 in NPI-NH)					
				(A) 22.73 ± 14.30			(Zopiclone (7.5–10 mg/day) and brotizolam (0.25 mg/day), if needed for insomnia)		
				(B1) 26.20 ± 15.77					
				(B2) 23.24 ± 15.53					
Terasawa et al. (1997)	139(69:70)	(A) 75.7 ± 8.9	(A) 69(28:41)	-VD (DSM-III-R)	NA	HM	Placebo	12 weeks/NR	1. TER (overall symptom)
	→119(55:64)	(B) 77.6 ± 7.9	(B) 70(22:48)	-Carlo Loeb modified ischemic score≥5					2. TER (subjective symptom)
									3. TER (neurological symptom)
									4. TER (psychiatric symptom)
									5. TER (ADL)
									6. TER (utility rating)
									7. HDS-R
Yao et al. (2014)	80(40:40)	(A) 71.3 ± 6.9	(A) 40(23:17)	-VD (Textbook in neurology)	internal obstruction of static blood	HM + (B)	Clopidogrel 75 mg/day	3 months/NR	1. ADL
	→80(40:40)	(B) 70.1 ± 8.1	(B) 40(21:19)	-CDR					2. CDR
				(A) 1.5 ± 0.5					3. HAMD
				(B) 1.5 ± 0.5					4. TER (ADL, CDR, HAMD)
				-HAMD					
				(A) 1.3 ± 0.6					
				(B) 1.4 ± 0.6					
Zhang (2012)	80(40:40)	(A) 74.55 ± 6.30	NR	-AD or VD (CCMD-3)	NA	HM	Haloperidol 1 mg/day (modification up to 4–10 mg/day within 2 weeks)	8 weeks/NR	1. TER (BEHAVE-AD)
	→80(40:40)	(B) 74.43 ± 6.45		-MMSE<24					2. BEHAVE-AD
				-BEHAVE-AD≥8					
				(A) 16.2 ± 7.8					
				(B) 16.1 ± 7.6					
Zhang et al. (2015b)	144(72:72)	(A) 72.79 ± 6.76	(A) 72(26:46)	-AD (DSM-IV)	NA	HM + Donepezil placebo	HM placebo + Donepezil	24 weeks/24 weeks	1. ADAS-cog
	→144(72:72)	(B) 72.97 ± 6.59	(B) 72(29:43)	-MMSE					2. MMSE
				(A) 20.49 ± 4.29					3. ADL
				(B) 19.82 ± 3.54					4. NPI
				-NPI					
				(A) 1.50 ± 2.96					
				(B) 1.35 ± 2.04					
				-HIS≤4					
				-HAMD≤7					
				-CDR 1					
Zhang et al. (2018)	94(47:47	(A) 67.2 ± 6.9	(A) 47(23:24)	-AD (NINCDS-ADRDA)	Liver depression and spleen deficiency	HM + (B)	Buspirone 15 mg/day (modification up to 30 mg/day), Sertraline 100 mg/day	6 weeks/NR	1. CSDD
	→94(47:47)	(B) 68.1 ± 6.9	(B) 47(22:25)	-CSDD>8					2. HAMA
				-HAMA>14					3. HAMD
				-HAMD>17					4. GQOLI-74
									5. TESS
Zhou (2015a)	40(20:20)	(A) 73.89 ± 4.31	(A) 18(7:11)	-AD (NINCDS-ADRDA)	Liver depression and spleen deficiency	HM + Huperzine A 200ug/day	Escitalopram 5 mg/day (modification up to 10 mg/day) + Huperzine A 200ug/day	6 weeks/NR	1. CSDD
	→36(18:18)	(B) 73.61 ± 3.73	(B) 18(6:12)	-Depression (NIMH-dAD, DSM-IV-TR)					2. TER (CSDD)
				-CSDD>8					3. SF-36
				(A) 15.44 ± 2.52					4. TCM symptom score
				(B) 15.11 ± 2.93					5. TER (TCM symptom score)
				-HIS≤4					
				-MMSE 10∼24					
				-CDR 1 or 2					
Zhou (2015b)	80(40:40)	(A) 54.5 ± 6.1	(A) 40(21:19)	-AD (DSM-IV)	NA	HM + Donepezil 5 mg/day	Escitalopram 10 mg/day + Donepezil 5 mg/day	3 months/NR	1. HAMD
	→80(40:40)	(B) 55.5 ± 6.7	(B) 40(19:21)	-MMSE					2. MMSE
				(A) 16.07 ± 2.44					
				(B) 16.77 ± 3.16					
				-Depression (CCMD-3)					
Zhou (2018)	80(40:40)	72.35 ± 3.24	80(44:36)	-AD (CCMD)	NA	HM	Donepezil 10 mg/day, Magnesium valproate sustained-release 500 mg/day	1 month/NR	1. TER (MMSE)
	→80(40:40)			-MMSE					2. MMSE
				(A) 22.93 ± 2.41					3. PSQI
				(B) 22.86 ± 2.27					
Zhou et al. (2019)	60(30:30)	(A) 62.20 ± 5.56	(A) 30(17:13)	-VD (NINDS-AIREN)	qi deficiency, phlegm, and stasis	HM + (B)	Donepezil 5 mg/day	8 weeks/NR	1. TER (TCM symptom score)
	→60(30:30)	(B) 63.32 ± 5.18	(B) 30(14:16)	-MMSE≤23					2. MMSE
				(A) 16.83 ± 2.10					3. ADL
				(B) 17.23 ± 2.43					4. NPI
				-NPI					5. TCM symptom score
				(A) 44.00 ± 13.83					6. Plasma hs-CRP
				(B) 46.47 ± 13.61					7. Plasma TNF-α
				-HIS≥7					8. Plasma IL-6
				-CDR (mild to moderate dementia)					9. Plasma Hcy
									10. Plasma MDA
									11. Plasma SOD
Zuo (2017)	56(30:26)	(A) 66.5 ± 12.3	(A) 30(25:5)	-AD (IWG-2 criteria)	NA	HM	Sertraline 50 mg/day (modification up to 100 mg/day)	60 days/NR	1. TER (HAMD)
	→56(30:26)	(B) 67.6 ± 10.8	(B) 26(20:6)	-Depression (HAMD≥17)					2. HAMD

ACE-R, Addenbrooke cognitive examination revised; AD, Alzheimer’s disease; ADAS-cog, Alzheimer's disease assessment scale–cognitive subscale; ADL, activities of daily living; BDNF, brain-derived neurotrophic factor; BEHAVE-AD, behavioral pathology in Alzheimer's disease rating scale; BPRS, brief psychiatric rating scale; CCMD, Chinese classification of mental disorders; CDR, clinical dementia rating; CDT, clock drawing test; CIBIC-plus, clinician’s interview-based impression of change-plus caregiver information; CRP, C-reactive protein; CSDD, Cornell scale for depression in dementia; DAD, disability assessment of dementia; DIEPSS, drug-induced extra-pyramidal symptoms scale; DLB, dementia with Lewy bodies; DSM, diagnostic and statistical manual of mental disorders; FIM, functional independence measure; GBS, Gottfries-Bråne-Steen; GDS, global deterioration scale; GQOLI-74, generic quality of life inventory-74; HAMA, Hamilton anxiety rating scale; HAMD, Hamilton depression rating scale; Hcy, homocysteine; HDS, Hasegawa's dementia scale; HDS-R, the revised Hasegawa's dementia scale; HIS, Hachinski ischemia score; HM, herbal medicine; IADL, instrumental activities of daily living; ICD, the international statistical classification of diseases and related health problems; IL, interleukin; IWG, international working group; MDA, malondialdehyde; MMSE, mini-mental state examination; MoCA, Montreal cognitive assessment; NA, not applicable; NIMH-dAD, National Institute of Mental Health criteria for depression in Alzheimer's disease; NINCDS-ADRDA, national institute of neurological and communicative diseases and stroke/Alzheimer's disease and related disorders association; NINDS-AIREN, national Institute of neurological disorders and stroke and association internationale pour la Recherché et l'Enseignement en neurosciences; NPI, neuropsychiatric inventory; NPI-NH, neuropsychiatric inventory-nursing home; NPI-Q, neuropsychiatric inventory-questionnaire; NR, not recorded; PSQI, Pittsburgh sleep quality index; SD, senile dementia; SDS, self-rating depression scale; SDSD, dementia syndrome type scale; SF-36, 36-item short form survey; SOD, superoxide dismutase; TCM, traditional Chinese medicine; TER, total effective rate; TESS, treatment emergent symptom scale; TMT, trail making test; TNF, tumor necrosis factor; VADAS-cog, vascular dementia assessment scale-cognitive subscale; VD, vascular dementia

### RoB in Studies

**FIGURE 2:**
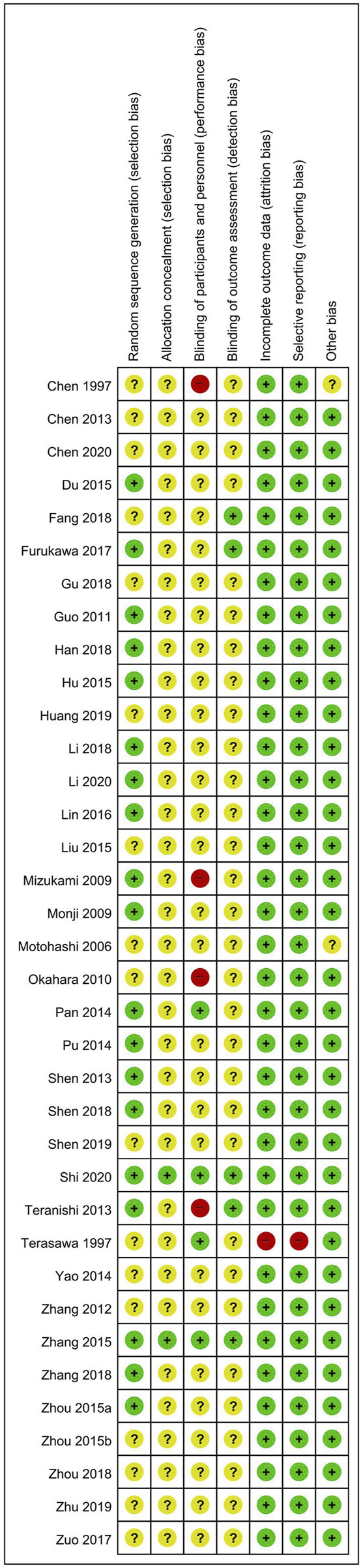
Risk of bias for all included studies. Low, unclear, and high risk, respectively, are represented with the following symbols: “+”, “?”, and “-”.

For RCTs, a total of 19 (Du et al., 2015; Furukawa et al., 2017; Guo et al., 2011a; Han, 2018; Hu et al., 2015; Li, 2020; Li et al., 2018; Lin et al., 2016; Mizukami et al., 2009; Monji et al., 2009; Pan et al., 2014; Pu et al., 2014; Shen, 2013; Shen et al., 2018; Shi et al., 2020; Teranishi et al., 2013; Zhang et al., 2015a; Zhang et al., 2018; Zhou, 2015) and two studies (Shi et al., 2020; Zhang et al., 2015a) were evaluated as having a low RoB in the corresponding domain, mentioning the appropriate random sequence generation method and allocation concealment, respectively. Each of four (Pan et al., 2014; Shi et al., 2020; Teranishi et al., 2013; Zhang et al., 2015a) and five studies (Fang et al., 2018; Furukawa et al., 2017; Shi et al., 2020; Teranishi et al., 2013; Zhang et al., 2015a) were evaluated with low risk of performance or detection bias by appropriately performing blinding of participants, personnel, or outcome assessors. In one study (Terasawa et al., 1997), the number of dropouts in each group was not described in detail, and the lack of outcomes related to BPSD was evaluated as a high risk of attrition and reporting bias. Two studies (Chen et al., 1997; Motohashi, 2006) were evaluated as having an unclear risk of other bias because there was no information on the homogeneity of baseline clinical characteristics between the two groups (Figure 2). In one CCT (Xu, 2018), the treatment or control group or outcome measures were not properly specified. In 12 before–after studies (Iwasaki et al., 2005; Xu et al., 2007; Shinno et al., 2008; Hayashi et al., 2010; Kawanabe et al., 2010; Guo et al., 2011b; Iwasaki et al., 2012; Nagata et al., 2012; Yang et al., 2012; Sumiyoshi et al., 2013; Ohsawa et al., 2017; Manabe, 2020), study questions, eligibility criteria for the study population, interventions, and outcome measures were clearly stated in most studies. However, blinding of outcome assessors was not reported in all studies, and only two studies (Iwasaki et al., 2005; Manabe, 2020) provided individual-level data (Supplementary Material S5).

### Effectiveness (or Efficacy) and Safety of HM in Included RCTs

#### HM as a Monotherapy

Compared to psychotropic drugs, the severity of BPSD symptoms evaluated by BEHAVE-AD was not significantly different between the two groups (MD = −0.36; 95% CI: −1.09 to 0.36), regardless of the treatment duration. However, as a result of subgroup analysis according to the severity and type of dementia, in the case of severe dementia and vascular dementia, BEHAVE-AD was significantly reduced in the HM group (MD = −2.34, 95% CI: −4.15 to −0.53). The Hamilton depression rating scale (HAMD) was significantly improved in the HM group (MD = −2.86; 95% CI: −3.85 to −1.87), but there was no significant difference between the two groups in TER calculated based on BPSD symptoms (RR = 1.05, 95% CI: 0.95–1.16). To evaluate the severity of BPSD, NPI (Zhang et al., 2015a), NPI-nursing home (Teranishi et al., 2013), and the Cornell scale for depression in dementia (CSDD) (Zhou, 2015) were evaluated in one each, and there was no significant difference between the two groups in all studies. In addition, AE occurred significantly less in the HM group (RR = 0.40, 95% CI: 0.25–0.64), regardless of the treatment duration (Table 2). However, when the sensitivity analysis was performed on studies with low risk of performance and detection bias, there was no significant difference in the meta-analysis of AE between the two groups (RR = 0.83; 95% CI: 0.27–2.61). Overall, severe AEs related to HM were rarely reported, but hypokalemia in HMs containing licorice, such as Yokukansan, required attention (Supplementary Material S6).

**TABLE 2:** Effect estimates of meta-analysis.

Outcomes	Subgroup	No. RCTs	No. participants	Effect estimate MD/RR (95%CI)	*I*^*2*^ value (%)	Model
**Herbal medicine vs. psychotropic drugs**						
BEHAVE-AD	Total	4	320	MD -0.36 [-1.09, 0.36]	45	Fixed
Dementia severity/type	Moderate/2 or more dementia	3	250	MD 0.01 [-0.78, 0.80]	0	Fixed
	Severe/VD	1	70	MD -2.34 [-4.15, -0.53]	NA	Fixed
Treatment duration	≤1 month	1	60	MD 0.20 [-3.17, 3.57]	NA	Fixed
	1 < month ≤ 2	2	150	MD -0.63 [-1.57, 0.31]	79	Fixed
	2 < month ≤ 6	1	110	MD 0.00 [-1.20, 1.20]	NA	Fixed
HAMD	Total (AD)	2	136	MD -2.86 [-3.85, -1.87]	87	Fixed
Dementia severity/treatment duration	Unclear/1 < month ≤ 2	1	56	MD -4.28 [-5.70, -2.86]	NA	Fixed
	Moderate/2 < month ≤ 6	1	80	MD -1.50 [-2.89, -0.11]	NA	Fixed
TER (BPSD symptom)	Total	5	332	RR 1.05 [0.95, 1.16]	45	Fixed
Dementia severity/type	Moderate/2 or more dementia	2	170	RR 0.95 [0.85, 1.06]	0	Fixed
	Severe/VD	1	70	RR 1.15 [0.93, 1.43]	NA	Fixed
	Unclear/AD	2	92	RR 1.22 [0.92, 1.61]	66	Fixed
Treatment duration	≤1 month	1	60	RR 0.96 [0.83, 1.12]	NA	Fixed
	1 < month ≤ 2	3	162	RR 1.18 [0.99, 1.41]	31	Fixed
	2 < month≤6	1	110	RR 0.94 [0.81, 1.09]	NA	Fixed
Adverse event	Total	4	360	RR 0.40 [0.25, 0.64]	0	Fixed
Dementia severity	Mild	1	144	RR 0.83 [0.27, 2.61]	NA	Fixed
	Moderate	1	110	RR 0.35 [0.15, 0.83]	NA	Fixed
	Severe	1	70	RR 0.35 [0.18, 0.67]	NA	Fixed
	Unclear	1	36	RR 0.20 [0.01, 3.89]	NA	Fixed
Dementia type	AD	2	180	RR 0.65 [0.23, 1.82]	0	Fixed
	VD	1	70	RR 0.35 [0.18, 0.67]	NA	Fixed
	2 or more	1	110	RR 0.35 [0.15, 0.83]	NA	Fixed
Treatment duration	1 < month ≤ 2	2	106	RR 0.33 [0.18, 0.63]	0	Fixed
	2 < month ≤ 6	2	254	RR 0.48 [0.25, 0.93]	28	Fixed
**Herbal medicine vs. placebo**						
Adverse event	Total	4	662	RR 2.67 [0.93, 7.65]	0	Fixed
Dementia severity	Mild	1	287	RR 0.95 [0.11, 8.32]	NA	Fixed
	Moderate	2	236	RR 5.60 [0.69, 45.36]	NA	Fixed
	Unclear	1	139	RR 2.54 [0.51, 12.63]	NA	Fixed
Dementia type	AD	2	236	RR 5.60 [0.69, 45.36]	NA	Fixed
	VD	2	426	RR 1.82 [0.52, 6.42]	0	Fixed
Treatment duration	≤1 month	1	145	RR 5.60 [0.69, 45.36]	NA	Fixed
	2 < month ≤ 6	3	517	RR 1.82 [0.52, 6.42]	0	Fixed
**Herbal medicine + psychotropic drugs vs. psychotropic drugs**						
BEHAVE-AD	Total	3	274	MD -3.48 [-3.96, -2.99]	87	Fixed
Dementia severity/treatment duration	Moderate/1 < month ≤ 2	2	174	MD -3.48 [-4.07, -2.89]	93	Fixed
	Unclear/2 < month ≤ 6	1	100	MD -3.47 [-4.30, -2.64]	NA	Fixed
Dementia type	AD	2	184	MD -2.96 [-3.52, -2.39]	63	Fixed
	SD	1	90	MD -4.90 [-5.83, -3.97]	NA	Fixed
TER (BPSD symptom)	Total	4	354	RR 1.16 [1.05, 1.29]	0	Fixed
Dementia severity/treatment duration	Moderate/1 < month ≤ 2	2	174	RR 1.19 [1.04, 1.37]	0	Fixed
	Unclear/2 < month ≤ 6	2	180	RR 1.13 [0.98, 1.31]	0	Fixed
Dementia type	AD	3	264	RR 1.16 [1.03, 1.30]	0	Fixed
	SD	1	90	RR 1.18 [0.96, 1.46]	NA	Fixed
Adverse event	Total	5	369	RR 0.71 [0.50, 0.99]	49	Fixed
Dementia severity	Moderate	3	189	RR 0.79 [0.48, 1.31]	49	Fixed
	Unclear	2	180	RR 0.64 [0.40, 1.01]	75	Fixed
Dementia type	AD	4	279	RR 0.62 [0.42, 0.92]	52	Fixed
	SD	1	90	RR 1.09 [0.54, 2.21]	NA	Fixed
Treatment duration	1 < month ≤ 2	2	174	RR 0.72 [0.43, 1.20]	62	Fixed
	2 < month ≤ 6	3	195	RR 0.70 [0.45, 1.09]	62	Fixed
**Herbal medicine + anti-dementia drugs vs. anti-dementia drugs**						
BEHAVE-AD	Total	4	333	MD -2.81 [-3.17, -2.45]	89	Fixed
Dementia severity	Moderate	2	168	MD -3.06 [-3.44, -2.68]	85	Fixed
	Unclear	2	165	MD -0.96 [-2.00, 0.08]	85	Fixed
Dementia type	AD	3	228	MD -3.04 [-3.42, -2.67]	70	Fixed
	2 or more	1	105	MD 0.10 [-1.22, 1.42]	NA	Fixed
Treatment duration	≤1 month	2	142	MD -3.20 [-3.60, -2.81]	0	Fixed
	2 < month ≤ 6	2	191	MD -0.84 [-1.72, 0.04]	72	Fixed
NPI	Total (Moderate)	4	264	MD -3.23 [-4.06, -2.40]	97	Fixed
Dementia type	VD	1	60	MD -5.76 [-10.67, -0.85]	NA	Fixed
	AD	3	204	MD -3.15 [-4.00, -2.31]	98	Fixed
Treatment duration	≤1 month	1	61	MD -5.40 [-12.48, 1.68]	NA	Fixed
	1 < month ≤ 2	1	60	MD -5.76 [-10.67, -0.85]	NA	Fixed
	2 < month ≤ 6	2	143	MD -3.12 [-3.97, -2.27]	99	Fixed
HAMD	Total (1 < month ≤ 2)	2	180	MD -4.92 [-5.48, -4.37]	83	Fixed
Dementia severity/type	Severe/VD	1	90	MD -5.30 [-5.94, -4.66]	NA	Fixed
	Unclear/AD	1	90	MD -3.70 [-4.85, -2.55]	NA	Fixed
TER (BPSD symptom)	Total	3	255	RR 1.29 [1.13, 1.47]	65	Fixed
Dementia severity	Severe	1	90	RR 1.28 [1.04, 1.58]	NA	Fixed
	Unclear	2	165	RR 1.29 [1.09, 1.54]	83	Fixed
Dementia type/treatment duration	AD/≤1 month	1	60	RR 1.80 [1.23, 2.62]	NA	Fixed
	VD/1 < month ≤ 2	1	90	RR 1.28 [1.04, 1.58]	NA	Fixed
	2 or more/2 < month ≤ 6	1	105	RR 1.11 [0.93, 1.33]	NA	Fixed
Barthel index	Total (2 < month ≤ 6)	2	162	MD 3.42 [2.67, 4.16]	99	Fixed
Dementia severity/type	Moderate/AD	1	57	MD 1.20 [0.34, 2.06]	NA	Fixed
	Unclear/2 or more	1	105	MD 10.44 [8.91, 11.97]	NA	Fixed
Adverse event	Total	5	376	RR 0.50 [0.28, 0.88]	15	Fixed
Dementia severity	Moderate	2	121	RR 2.00 [0.19, 20.90]	NA	Fixed
	Severe	1	90	RR 0.31 [0.11, 0.87]	NA	Fixed
	Unclear	2	165	RR 0.56 [0.27, 1.16]	26	Fixed
Dementia type/treatment duration	AD/≤1 month	2	121	RR 2.00 [0.19, 20.90]	NA	Fixed
	VD/1 < month ≤ 2	2	150	RR 0.43 [0.17, 1.05]	51	Fixed
	2 or more/2 < month ≤ 6	1	105	RR 0.46 [0.21, 1.03]	NA	Fixed

AD, Alzheimer’s disease; BEHAVE-AD, behavioral pathology in Alzheimer’s disease rating scale; BPSD, behavioral and psychological symptoms of dementia; CI, confidence interval; HAMD, Hamilton depression rating scale; MD, mean difference; NA, not applicable; NPI, neuropsychiatric inventory; RCT, randomized controlled trial; RR, risk ratio; SD, senile dementia; TER, total effective rate; VD, vascular dementia.

When comparing HM and placebo, in one study (Furukawa et al., 2017), there was no significant difference between the two groups in the NPI-questionnaire. However, in another study (Pan et al., 2014) that measured the severity of BPSD symptoms, the HM group showed significant improvement in hallucinations, activity disturbances, aggressiveness, and anxieties and phobias of BEHAVE-AD, and in delusions, hallucinations, agitation, aberrant motor behavior, and sleep disturbance of NPI (*p* < 0.05), although there were no significant differences between the two groups in other domains. In a study comparing the group administered HM and donepezil placebo with the group administered with HM placebo and donepezil placebo (Shi et al., 2020), it was reported that the change in the NPI total score after treatment was significantly greater in the group administered with HM and donepezil placebo (*p* < 0.05). Finally, a study that evaluated TER based on psychiatric symptoms reported that TER of the HM group was significantly higher than that of the placebo group (Terasawa et al., 1997). There was no significant difference in the incidence of AE between the two groups (RR = 2.67; 95% CI: 0.93–7.65) (Table 2). Even when the sensitivity analysis was performed only with studies with a low risk of performance and detection bias, this result was not affected.

When comparing HM and anti-dementia drugs, one study (Zhou, 2018) showed that the Pittsburgh Sleep Quality Index (PSQI) was significantly reduced in the HM group (*p* < 0.05). In one study comparing HM and no treatment (Mizukami et al., 2009), the severity of BPSD symptoms measured by NPI after 4 weeks of treatment was significantly improved in the HM group (*p* < 0.05), but the Barthel index or IADL was not significantly different between the two groups. In one study comparing HM and hydergine (Chen et al., 1997), there was no significant difference in the HAMD score after treatment.

#### HM as an Adjunctive Therapy

When HM was additionally used for psychotropic drugs, the BEHAVE-AD (MD = −3.48, 95% CI: −3.96 to −2.99) and TER calculated based on BPSD symptoms (RR = 1.16, 95% CI: 1.05–1.29) significantly improved, compared with psychotropic drugs alone. In addition, the incidence of AE was also significantly lower in the HM group (RR = 0.71; 95% CI: 0.50–0.99; Table 2). When HM was additionally used, one study Gu et al. (2018) reported that the BPRS score improved significantly (*p* < 0.05), and another study Zhang et al. (2018) reported that QoL and the severity of BPSD evaluated by CSDD, Hamilton Anxiety Rating Scale, and HAMD significantly improved, and the frequency of side effects evaluated by treatment emergent symptom scale was significantly reduced (*p* < 0.05, all).

When HM was additionally used as an anti-dementia drug, the severity of BPSD symptoms measured by BEHAVE-AD (MD = −2.81, 95% CI: −3.17 to −2.45), NPI (MD = −3.23, 95% CI: −4.06 to −2.40), and HAMD (MD = −4.92, 95% CI: −5.48 to −4.37) significantly improved, compared with anti-dementia drugs alone. The TER calculated based on BPSD symptoms (RR = 1.29, 95% CI: 1.13–1.47) and Barthel index (MD = 3.42, 95% CI: 2.67–4.16) also significantly improved in the HM group. The severity of BPSD symptoms was evaluated using PSQI(30) (*p* < 0.05) and NPI-questionnaire (Hu et al., 2015) (*p* < 0.01) in each study, and both showed significantly improved results in the HM group. In one study (Okahara et al., 2010), the degree of depression and burden of caregivers after treatment were reported through a self-rating depression scale and a Zarit burden interview, respectively, but there was no significant difference between the two groups. The frequency of AE was also significantly lower in the HM group (RR = 0.50, 95% CI: 0.28–0.88), although there was no consistent result according to the subgroups (Table 2,Supplementary Material S6).

Furthermore, when HM was additionally used, there was no significant difference in the CSDD score compared with the oxiracetam alone (Shen et al., 2019). However, HAMD significantly improved (*p* < 0.05) when HM was additionally used to clopidogrel in one study (Yao et al., 2014).

### Publication Bias

Since there was no meta-analysis that included more than ten studies, we could not assess the publication bias using a funnel plot.

### Results From Other Included Studies

In addition to the included RCTs, all other studies (Iwasaki et al., 2005; Shinno et al., 2007; Xu et al., 2007; Shinno et al., 2008; Hayashi et al., 2010; Kawanabe et al., 2010; Guo et al., 2011b; Iwasaki et al., 2012; Nagata et al., 2012; Yang et al., 2012; Sumiyoshi et al., 2013; Kudoh et al., 2016; Ohsawa et al., 2017; Meguro and Yamaguchi, 2018; Xu, 2018; Manabe, 2020) have reported that HM improved BPSD in at least one indicator. However, considering the design of the study, these studies were not included as evidence for analyzing the effectiveness or efficacy of HM for BPSD but reviewed in terms of the current status of research in this field. More information can be found in Supplementary Material S3, S6.

## Discussion

### Summary of Evidence

In this systematic review, the most comprehensive review and meta-analysis to date was conducted to analyze the effectiveness (or efficacy), safety, and research status of HM for BPSD. According to the meta-analysis, HM did not show statistically significant differences from psychotropic drugs in the effectiveness of BPSD evaluated as BEHAVE-AD or TER or in the subgroup analysis of dementia severity, dementia type, or treatment duration. However, a few studies reported that HM showed statistically significant improvement in patients with Alzheimer’s disease compared to psychotropic drugs in HAMD. In addition, HM appeared to be safer compared to psychotropic drugs in terms of the incidence of AEs. Comparisons between HM and placebo in four studies did not have homogeneous outcomes in the meta-analysis. Of the four studies, three showed significant differences between the HM and placebo groups in improving BPSD symptoms but not in the one remaining study. The incidence of AE was not significantly different between the groups, and these results did not change according to the subgroup analysis on dementia severity, dementia type, or treatment duration. When HM was compared to anti-dementia drugs or no treatment, there were statistically significant benefits in improving sleep quality assessed by PSQI or BPSD symptoms assessed by NPI in a study, respectively. When HM was used as an adjunctive therapy, it showed the most consistent benefit. When HM was used in combination with psychotropic or anti-dementia drugs, there were statistically significant benefits in BEHAVE-AD, NPI, HAMD, TER based on BPSD symptoms, incidence of AEs, and Barthel index, compared to monotherapy with psychotropic or anti-dementia drugs. The methodological quality of the RCTs included in this systematic review was not optimal overall. In particular, allocation concealment and blinding domains were evaluated as unclear in most studies.

### Clinical Implications

HM is an EATM modality that has long been used in Asian countries for health improvement and disease treatment. The results of this systematic review and meta-analysis provided limited evidence that HM may be associated with additional benefits in BPSD treatment, particularly when used as an adjunct to conventional medications, including psychotropic and anti-dementia drugs. Although the clinical evidence supporting the effectiveness (or efficacy) and safety of HM for BSPD is insufficient, this topic has clinical relevance considering that many elderly patients already use prescription drugs and HM in combination (de Souza Silva et al., 2014; Agbabiaka et al., 2017). Additionally, the use of HMs is not limited to EATM. Herbs used in other traditions, such as *Ginkgo biloba*, *Withania somnifera*, *Panax ginseng*, and *Curcuma longa* *,* and some phytochemicals have also shown promising results in the treatment of dementia (Alzobaidi et al., 2021). Currently, studies are focused on drug delivery, such as improving the potential anti-dementia effect of HM by using a targeted nanocarrier system (Moradi et al., 2020; Singh et al., 2021). Any current or future studies that explore the therapeutic potential of HM for dementia should be encouraged, as these can provide valuable insight in the field.

Although not within the scope of this review, HM is also used to delay cognitive decline, a core symptom of dementia, and its mechanisms are being studied to be related to mechanisms such as anti-inflammatory, antioxidative, and antiapoptotic activity (Tewari et al., 2018). However, the underlying mechanism of HM for the core and associated symptoms of dementia is yet unclear, and it may be related to some challenges including non-uniform chemical composition, non-standardized ratio of herb ingredients, and its multi-component and multi-target mechanism (Zhou et al., 2019). Moreover, there are safety issues associated with HM, such as lack of safety monitoring and potential interactions with conventional pharmaceuticals (Ekor, 2014). Fortunately, for some standardized HMs, such as Yokukansan, underlying therapeutic mechanisms for dementia (Takeyoshi et al., 2016), potential interactions with conventional medications (Soraoka et al., 2016) and safety issues have been documented (Shimada et al., 2017). Similarly, a database of some potential herb-drug interactions relevant to the management of cognitive impairment has been recently developed. It provides the pharmacological interactions of 170 bio-actives with 10 commonly-used drugs (Auxtero et al., 2021). However, other heterogeneous HMs are also used in clinical practice and their safety profiles need further clarification. Spontaneous reporting systems and active pharmacovigilance for the use of HMs should be encouraged with stringent oversight by a national-level regulatory body to ensure patient safety and satisfaction (Zhang et al., 2015b).

In summary, in order for HM to be seamlessly integrated into the conventional medical system in the management of dementia, particularly to treat BPSD, the use of standardized HM with well-managed quality should be encouraged, and the underlying mechanisms and possible interactions with conventional pharmaceuticals should be further investigated. It should also be used by health care professionals in clinics or hospital-based settings for meticulous effectiveness and safety monitoring.

### Strengths and Limitations

This systematic review comprehensively reviewed the studies published to date on this issue and summarized the clinical evidence supporting the effectiveness and safety of HM in the management of BPSD. Considering the limitations of psychotropic drugs in the management of BPSD, particularly in the elderly, and many elderly patients already use HM, this topic has great clinical relevance. Our study highlights the limited evidence of HM for BPSD management and discusses the future directions necessary for HM to be integrated into conventional dementia care systems as an adjuvant therapy.

The findings of this systematic review should be interpreted with careful consideration of some limitations (Wimo et al., 2017). Although this review collected clinical evidence of HM for BPSD as the most comprehensive, the number of studies included in each meta-analysis was less than six because the studies included were heterogeneous. In particular, some standardized HMs, such as Yokukansan, existed, but most studies used HMs of heterogeneous composition. Although EATM is a medicine system that emphasizes holistic and individualized approaches (Fung and Linn, 2015), the use of standardized HM is emphasized in order to establish an effective HM use strategy for BPSD treatment and to confirm its expected effectiveness and safety. In addition, in order to accumulate robust clinical evidence of HM for BPSD management, the design of dementia severity, dementia type, BPSD severity, and treatment duration of subjects should be homogeneous (Ohno et al., 2019). In the protocol of this review (Kwon et al., 2021), the subgroup analysis was planned according to the severity of baseline BPSD of participants, but this subgroup analysis was not possible because of the heterogeneity of the indicators. However, since psychotropic drugs, such as antipsychotics, are generally more recommended for severe BPSD compared to safe non-pharmacological therapy (Masopust et al., 2018), finding other safe alternatives, including HM, in patients with severe BPSD, is necessary (Cerejeira et al., 2012). Since only a few studies were included in each meta-analysis, evaluation of publication bias through funnel plots was not possible. However, most studies included in the analysis were conducted and reported in China, which suggests a potential publication bias in the results. Although HM is mainly used in Asian countries as an EATM modality, rigorous clinical trials conducted in Taiwan and Korea, in addition to China and Japan, are encouraged to address this issue (van der Linde et al., 2016). None of the included studies reported the results of economic value related to HM for BPSD. Dementia causes a huge socioeconomic burden worldwide, and BPSD is a major contributing factor (Cerejeira et al., 2012). Therefore, effective alternatives to BPSD in the future require cost-effectiveness, effectiveness, and safety. Considering that the cost-effectiveness of HM is being studied for other clinical topics, such as chronic low back pain (Sung et al., 2019), further clinical research on HM for BPSD should encompass economic evaluation.

## Conclusion

According to the findings of this review, HM may be associated with additional benefits in BPSD treatment, particularly when used as an adjunct to conventional medications, including psychotropic and anti-dementia drugs. However, considering the methodological quality of the included RCTs, this clinical evidence is not robust. In addition, the heterogeneity of HMs used in each study encourages the use of standardized HMs in the future. Nevertheless, dementia is a global health concern, and considering the limitations of conventional psychotropic drugs for BPSD, a major cause of the disease burden, HM appears to be a promising complementary therapy that warrants further research.

## Data Availability

The original contributions presented in the study are included in the article/Supplementary Material, further inquiries can be directed to the corresponding author.

## References

[B1] Agbabiaka T. B. Wider B. Watson L. K. Goodman C. (2017). Concurrent Use of Prescription Drugs and Herbal Medicinal Products in Older Adults: A Systematic Review. Drugs Aging 34 (12), 891–905. 10.1007/s40266-017-0501-7 29196903 PMC5730633

[B2] Alzobaidi N. Quasimi H. Emad N. A. Alhalmi A. Naqvi M. (2021). Bioactive Compounds and Traditional Herbal Medicine: Promising Approaches for the Treatment of Dementia. Degener Neurol. Neuromuscul. Dis. 11, 1–14. 10.2147/DNND.S299589 33880073 PMC8051957

[B3] Auxtero M. D. Chalante S. Abade M. R. Jorge R. Fernandes A. I. (2021). Potential Herb-Drug Interactions in the Management of Age-Related Cognitive Dysfunction. Pharmaceutics 13 (1), 124. 10.3390/pharmaceutics13010124 33478035 PMC7835864

[B4] Balshem H. Helfand M. Schünemann H. J. Oxman A. D. Kunz R. Brozek J. (2011). GRADE Guidelines: 3. Rating the Quality of Evidence. J. Clin. Epidemiol. 64 (4), 401–406. 10.1016/j.jclinepi.2010.07.015 21208779

[B5] By the 2019 American Geriatrics Society Beers Criteria® Update Expert Panel (2019). American Geriatrics Society 2019 Updated AGS Beers Criteria® for Potentially Inappropriate Medication Use in Older Adults. J. Am. Geriatr. Soc. 67 (4), 674–694. 10.1111/jgs.15767 30693946

[B6] Cerejeira J. Lagarto L. Mukaetova-Ladinska E. B. (2012). Behavioral and Psychological Symptoms of Dementia. Front. Neurol. 3, 73. 10.3389/fneur.2012.00073 22586419 PMC3345875

[B7] Chen K. Chen K. J. Zhou W. Q. (1997). [Clinical Study of Effect of Yizhi Capsule on Senile Vascular Dementia]. Zhongguo Zhong Xi Yi Jie He Za Zhi 17 (7), 393–397. 10322856

[B8] Chen T. Gao M. Liang H. (2013). Clinical Observation of Naoling Granules in Treating Behavioral and Mental Symptoms of Dementia. Yunnan J. Traditional Chin. Med. Materia Med. 34 (10), 33–34. 10.16254/j.cnki.53-1120/r.2013.10.005

[B9] Chen Y. (2020). Effect of Yangxue Qingnao Granules on Sleep Quality of Patients with Senile Dementia and Insomnia. Chin. J. Integr. Med. Cardio-/Cerebrovascuiar Dis. 18 (20), 3469–3471. 10.12102/j.issn.1672-1349.2020.20.041

[B10] Cummings J. L. Mega M. Gray K. Rosenberg-Thompson S. Carusi D. A. Gornbein J. (1994). The Neuropsychiatric Inventory: Comprehensive Assessment of Psychopathology in Dementia. Neurology 44 (12), 2308–2314. 10.1212/wnl.44.12.2308 7991117

[B11] de Souza Silva J. E. Santos Souza C. A. da Silva T. B. Gomes I. A. Brito Gde. C. de Souza Araújo A. A. (2014). Use of Herbal Medicines by Elderly Patients: A Systematic Review. Arch. Gerontol. Geriatr. 59 (2), 227–233. 10.1016/j.archger.2014.06.002 25063588

[B12] Du G. Li H. Liu D. Hou Y. (2015). Application and Effect of Liuwei Dihuang Pills in Adjuvant Treatment of Alzheimer's Disease. Hebei Med. J. 37 (11), 1661–1663. 10.3969/j.issn.1002-7386.2015.11.020

[B13] Ekor M. (2014). The Growing Use of Herbal Medicines: Issues Relating to Adverse Reactions and Challenges in Monitoring Safety. Front. Pharmacol. 4, 177. 10.3389/fphar.2013.00177 24454289 PMC3887317

[B14] Fang J. Li X. Chen W. (2018). Effect of Shugan Jieyu Capsule on 5-HT and Dopamine Levels in Elderly Patients with Alzheimer's Disease and Depression. Pract. Geriatr. 32 (10), 946–949. 10.3969/j.issn.1003-9198.2018.10.013

[B15] Fung F. Y. Linn Y. C. (2015). Developing Traditional Chinese Medicine in the Era of Evidence-Based Medicine: Current Evidences and Challenges. Evid. Based Complement. Alternat Med. 2015, 425037. 10.1155/2015/425037 25949261 PMC4407626

[B16] Furukawa K. Tomita N. Uematsu D. Okahara K. Shimada H. Ikeda M. (2017). Randomized Double-Blind Placebo-Controlled Multicenter Trial of Yokukansan for Neuropsychiatric Symptoms in Alzheimer's Disease. Geriatr. Gerontol. Int. 17 (2), 211–218. 10.1111/ggi.12696 26711658

[B17] Galasko D. Bennett D. A. Sano M. Marson D. Kaye J. Edland S. D. (2006). ADCS Prevention Instrument Project: Assessment of Instrumental Activities of Daily Living for Community-Dwelling Elderly Individuals in Dementia Prevention Clinical Trials. Alzheimer Dis. Assoc. Disord. 20 (4 Suppl. 3), S152–S169. 10.1097/01.wad.0000213873.25053.2b 17135809

[B18] Gu J. Luo H. Zhang Z. (2018). The Effects of Liuwei Dihuang Wan Plus Olanzapine on Psychiatric Symptoms of Alzheimer's Patients. Clin. J. Chin. Med. 10 (14), 66–67. 10.3969/j.issn.1674-7860.2018.14.029

[B19] Guo Z. Chen X. Xing B. (2011). Clinical Study on Nourishing Yin and Soothing Liver Therapy in Treating Senile Dementia with Mental and Behavioral Abnormalities. J. Front. Med. 1 (24), 208–209. 10.3969/j.issn.2095-1752.2011.24.288

[B20] Guo Z. Chen X. Xing B. Luo S. Shen Y. (2011). Zhibaidihuang Decoction Combined with Donepezil in the Treatment of 30 Cases of Senile Dementia with Abnormal Mental Behavior. Zhejiang J. Integrated Traditional Chin. West. Med. 21 (7), 471–472. 10.3969/j.issn.1005-4561.2011.07.012

[B21] Guyatt G. Rennie D. Meade M. Cook D. (2002). Users' Guides to the Medical Literature: A Manual for Evidence-Based Clinical Practice. IL, United States: AMA press Chicago.

[B22] Han M. (2018). Clinical Observation on Jianwei Yunao Decoction in Treating for Alzheimer's Disease with Qi and Blood Deficiency Syndrome. Acta Chin. Med. 33 (5), 878–881. 10.16368/j.issn.1674-8999.2018.05.209

[B23] Hayashi Y. Ishida Y. Inoue T. Udagawa M. Takeuchi K. Yoshimuta H. (2010). Treatment of Behavioral and Psychological Symptoms of Alzheimer-type Dementia with Yokukansan in Clinical Practice. Prog. Neuropsychopharmacol. Biol. Psychiatry 34 (3), 541–545. 10.1016/j.pnpbp.2010.02.016 20184936

[B24] Higgins J. P. T. A. D. (2011). The Cochrane Collaboration. Chapter 8: Assessing Risk of Bias in Included Studies. Available at: http://www.cochrane-handbook.org.

[B25] Howes M. R. Fang R. Houghton P. J. (2017). Effect of Chinese Herbal Medicine on Alzheimer's Disease. Int. Rev. Neurobiol. 135, 29–56. 10.1016/bs.irn.2017.02.003 28807163

[B26] Hu X. J. Yu C. J. Li J. Wang Y. Zhou J. B. Cheng W. (2015). Clinical Analysis of Bushen Tongluo Decoction in Treating 40 Patients with Alzheimer Disease. Chin. J. Exp. Traditional Med. Formulae 21 (11), 182–185. 10.13422/j.cnki.syfjx.2015110182

[B27] Huang Q. Xu Z. (2019). Observation on Curative Effect of Bushen Jiannao Decoction Combined with Olanzapine in Treating Mental Behavior Disorders and Senile Dementia. Mod. J. Integrated Traditional Chin. West. Med. 28 (24), 2701–2703. 10.3969/j.issn.1008-8849.2019.24.020

[B28] Iwasaki K. Kosaka K. Mori H. Okitsu R. Furukawa K. Manabe Y. (2012). Improvement in Delusions and Hallucinations in Patients with Dementia with Lewy Bodies upon Administration of Yokukansan, A Traditional Japanese Medicine. Psychogeriatrics 12 (4), 235–241. 10.1111/j.1479-8301.2012.00413.x 23279145

[B29] Iwasaki K. Maruyama M. Tomita N. Furukawa K. Nemoto M. Fujiwara H. (2005). Effects of the Traditional Chinese Herbal Medicine Yi-Gan San for Cholinesterase Inhibitor-Resistant Visual Hallucinations and Neuropsychiatric Symptoms in Patients with Dementia with Lewy Bodies. J. Clin. Psychiatry 66 (12), 1612–1613. 10.4088/JCP.v66n1219a 16401166

[B30] Kasper J. D. Black B. S. Shore A. D. Rabins P. V. (2009). Evaluation of the Validity and Reliability of the Alzheimer Disease-Related Quality of Life Assessment Instrument. Alzheimer Dis. Assoc. Disord. 23 (3), 275–284. 10.1097/WAD.0b013e31819b02bc 19812471 PMC3086660

[B31] Kawanabe T. Yoritaka A. Shimura H. Oizumi H. Tanaka S. Hattori N. (2010). Successful Treatment with Yokukansan for Behavioral and Psychological Symptoms of Parkinsonian Dementia. Prog. Neuropsychopharmacol. Biol. Psychiatry 34 (2), 284–287. 10.1016/j.pnpbp.2009.11.019 19948198

[B32] Kudoh C. Arita R. Honda M. Kishi T. Komatsu Y. Asou H. (2016). Effect of Ninjin'yoeito, a Kampo (Traditional Japanese) Medicine, on Cognitive Impairment and Depression in Patients with Alzheimer's Disease: 2 Years of Observation. Psychogeriatrics 16 (2), 85–92. 10.1111/psyg.12125 25918972

[B33] Kwon C. Y. Lee B. Ha D. J. (2021). Herbal Medicine for Behavioral and Psychological Symptoms of Dementia: a Protocol for Systematic Review. Medicine (Baltimore) 100 (8), e24577. 10.1097/md.0000000000024577 33663066 PMC7909178

[B34] Landi F. Onder G. Cesari M. Barillaro C. Russo A. Bernabei R. (2005). Psychotropic Medications and Risk for Falls Among Community-Dwelling Frail Older People: an Observational Study. J. Gerontol. A. Biol. Sci. Med. Sci. 60 (5), 622–626. 10.1093/gerona/60.5.622 15972615

[B35] Li Q. (2020). Effect of Bushan Zhuangshen Recipe on Cognitive Function and Plasma CRP and Hcy Levels in Alzheimer's Disease. Guangming J. Chin. Med. 35 (22), 3577–3579. 10.3969/j.issn.1003-8914.2020.22.032

[B36] Li W. Huang S. X. Zhu Y. P. (2018). Clinical Effect of Bushen Yizhi Formula Combined with Clozapine on Alzheimer's Disease Complicated with Behavior Disorders. Guangxi Med. J. 40 (22), 2682–2684. 10.11675/j.issn.0253-4304.2018.22.14

[B37] Lin Y. Chu W. Tang Y. (2016). Clinical Observation of Aripiprazole Combined with Fufang Haishe Capsule in Treating Alzheimer's Disease. J. New Chin. Med. 48 (12), 26–27. 10.13457/j.cnki.jncm.2016.12.011

[B38] Linacre J. M. Heinemann A. W. Wright B. D. Granger C. V. Hamilton B. B. (1994). The Structure and Stability of the Functional Independence Measure. Arch. Phys. Med. Rehabil. 75 (2), 127–132. 10.1016/0003-9993(94)90384-0 8311667

[B39] Liu S. Di G. He W. (2015). Curative Effect Observation of Bushen Yizhi Granule Combined with Donepezil Hydrochloride Dispersible Tablets in the Treatment of Alzheimer's Disease. Hebei Med. J. 37 (17), 2649–2651. 10.3969/j.issn.1002-7386.2015.17.030

[B40] Mahoney F. I. Barthel D. W. (1965). Functional Evaluation: the Barthel index. Md. State. Med. J. 14, 61–65. 14258950

[B41] Manabe Y. (2020). A Preliminary Trial in the Efficacy of Yokukansankachimpihange on REM Sleep Behavior Disorder in Dementia with Lewy Bodies. Front. Nutr. 7, 119. 10.3389/fnut.2020.00119 32923452 PMC7456844

[B42] Masopust J. Protopopová D. Vališ M. Pavelek Z. Klímová B. (2018). Treatment of Behavioral and Psychological Symptoms of Dementias with Psychopharmaceuticals: a Review. Neuropsychiatr. Dis. Treat. 14, 1211–1220. 10.2147/NDT.S163842 29785112 PMC5953267

[B43] Matsuda Y. Kishi T. Shibayama H. Iwata N. (2013). Yokukansan in the Treatment of Behavioral and Psychological Symptoms of Dementia: a Systematic Review and Meta-Analysis of Randomized Controlled Trials. Hum. Psychopharmacol. 28 (1), 80–86. 10.1002/hup.2286 23359469

[B44] Meguro K. Yamaguchi S. (2018). Decreased Behavioral Abnormalities after Treatment with Combined Donepezil and Yokukansankachimpihange in Alzheimer Disease: an Observational Study. The Osaki-Tajiri Project. Neurol. Ther. 7 (2), 333–340. 10.1007/s40120-018-0109-9 30255467 PMC6283791

[B45] Mizukami K. Asada T. Kinoshita T. Tanaka K. Sonohara K. Nakai R. (2009). A Randomized Cross-Over Study of a Traditional Japanese Medicine (Kampo), Yokukansan, in the Treatment of the Behavioural and Psychological Symptoms of Dementia. Int. J. Neuropsychopharmacol. 12 (2), 191–199. 10.1017/S146114570800970X 19079814

[B46] Monji A. Takita M. Samejima T. Takaishi T. Hashimoto K. Matsunaga H. (2009). Effect of Yokukansan on the Behavioral and Psychological Symptoms of Dementia in Elderly Patients with Alzheimer's Disease. Prog. Neuropsychopharmacol. Biol. Psychiatry 33 (2), 308–311. 10.1016/j.pnpbp.2008.12.008 19138715

[B47] Moradi S. Z. Momtaz S. Bayrami Z. Farzaei M. H. Abdollahi M. (2020). Nanoformulations of Herbal Extracts in Treatment of Neurodegenerative Disorders. Front. Bioeng. Biotechnol. 8, 238. 10.3389/fbioe.2020.00238 32318551 PMC7154137

[B48] Motohashi K. (2006). Clinical and Basic Research on the Influence of Guanyuan Granules on Cognitive Dysfunction and Behavioral-Psychological Symptoms of Vascular Dementia [Master's Degree]. Beijing: Beijing University of Chinese Medicine.

[B49] Nagata K. Yokoyama E. Yamazaki T. Takano D. Maeda T. Takahashi S. (2012). Effects of Yokukansan on Behavioral and Psychological Symptoms of Vascular Dementia: an Open-Label Trial. Phytomedicine 19 (6), 524–528. 10.1016/j.phymed.2012.02.008 22421528

[B50] National Heart, Lung, and Blood Institute (NHLBI) (2013). Study Quality Assessment Tools. MD, United States: National Institutes of Health. Available at: NHLBI website [Internet] https://www.nhlbi.nih.gov/health-topics/study-quality-assessment-tools.

[B51] Novak M. Guest C. (1989). Application of a Multidimensional Caregiver burden Inventory. Gerontologist 29 (6), 798–803. 10.1093/geront/29.6.798 2516000

[B52] Ohno Y. Kunisawa N. Shimizu S. (2019). Antipsychotic Treatment of Behavioral and Psychological Symptoms of Dementia (BPSD): Management of Extrapyramidal Side Effects. Front. Pharmacol. 10, 1045. 10.3389/fphar.2019.01045 31607910 PMC6758594

[B53] Ohsawa M. Tanaka Y. Ehara Y. Makita S. Onaka K. (2017). A Possibility of Simultaneous Treatment with the Multicomponent Drug, Ninjin'yoeito, for Anorexia, Apathy, and Cognitive Dysfunction in Frail Alzheimer's Disease Patients: an Open-Label Pilot Study. J. Alzheimers Dis. Rep. 1 (1), 229–235. 10.3233/ADR-170026 30480240 PMC6159634

[B54] Okahara K. Ishida Y. Hayashi Y. Inoue T. Tsuruta K. Takeuchi K. (2010). Effects of Yokukansan on Behavioral and Psychological Symptoms of Dementia in Regular Treatment for Alzheimer's Disease. Prog. Neuropsychopharmacol. Biol. Psychiatry 34 (3), 532–536. 10.1016/j.pnpbp.2010.02.013 20170698

[B55] Overall J. E. Gorham D. R. (1962). The Brief Psychiatric Rating Scale. Psychol. Rep. 10 (3), 799–812. 10.2466/pr0.1962.10.3.799

[B56] Ozaki T. Katsumata Y. Arai A. (2017). The Use of Psychotropic Drugs for Behavioral and Psychological Symptoms of Dementia Among Residents in Long-Term Care Facilities in Japan. Aging Ment. Health 21 (12), 1248–1255. 10.1080/13607863.2016.1220922 27584047

[B57] Page M. J. McKenzie J. E. Bossuyt P. M. Boutron I. Hoffmann T. C. Mulrow C. D. (2021). The PRISMA 2020 Statement: an Updated Guideline for Reporting Systematic Reviews. BMJ 372, n71. 10.1136/bmj.n71 33782057 PMC8005924

[B58] Pan W. Wang Q. Kwak S. Song Y. Qin B. Wang M. (2014). Shen-Zhi-Ling Oral Liquid Improves Behavioral and Psychological Symptoms of Dementia in Alzheimer's Disease. Evid. Based Complement. Alternat Med. 2014, 913687. 10.1155/2014/913687 24959193 PMC4052178

[B59] Park H. L. Lee H. S. Shin B. C. Liu J. P. Shang Q. Yamashita H. (2012). Traditional Medicine in china, Korea, and Japan: a Brief Introduction and Comparison. Evid. Based Complement. Alternat Med. 2012, 429103. 10.1155/2012/429103 23133492 PMC3486438

[B60] Pu Z. Fei Y. Lin Y. Xia J. (2014). Treatment of Vascular Dementia Patients with Agitation of Blood Stagnation Syndrome by Oxcarbazepine and Tongqiaohuoxue Decoction Separately: an Efficacy Comparison. Zhejiang J. Integrated Traditional Chin. West. Med. 24 (8), 659–661.

[B61] Ralph S. J. Espinet A. J. (2018). Increased All-Cause Mortality by Antipsychotic Drugs: Updated Review and Meta-Analysis in Dementia and General Mental Health Care. J. Alzheimers Dis. Rep. 2 (1), 1–26. 10.3233/adr-170042 30480245 PMC6159703

[B62] Sclan S. G. Saillon A. Franssen E. Hugonot-Diener L. Saillon A. Reisberg B. (1996). The Behavior Pathology in Alzheimer's Disease Rating Scale (Behave-Ad): Reliability and Analysis of Symptom Category Scores. Int. J. Geriat. Psychiatry 11 (9), 819–830. 10.1002/(sici)1099-1166(199609)11:9<819::aid-gps389>3.0.co;2-s

[B63] Shen Y. Chen S. Yu G. Yang H. (2019). Comparative Analysis of Clinical Efficacy of Xiaoyao Pills and Escitalopram in Treatment of Vascular Dementia Patients with Depression. Chin. Arch. Traditional Chin. Med. 37 (2), 396–399. 10.13193/j.issn.1673-7717.2019.02.034

[B64] Shen Y. (2013). Comparison of the Effects of Liuwei Dihuang Wan and Olanzapine on Improving the Mental and Behavioral Symptoms of Senile Dementia. Guiding J. Traditional Chin. Med. Pharm. 19 (12), 39–41. 10.13862/j.cnki.cn43-1446/r.2013.12.021

[B65] Shen Y. Yu G. Zhang H. (2018). Clinical Observation of Xiaoyao Pills Combined with Donepezil Hydrochloride in Treatment of Vascular Dementia Complicated with Depression. Chin. Arch. Traditional Chin. Med. 36 (7), 1724–1726. 10.13193/j.issn.1673-7717.2018.07.051

[B66] Shi J. Wei M. Ni J. Sun F. Sun L. Wang J. (2020). Tianzhi Granule Improves Cognition and BPSD of Vascular Dementia: a Randomized Controlled Trial. J. Transl Med. 18 (1), 76–10. 10.1186/s12967-020-02232-z 32054507 PMC7017567

[B67] Shimada S. Arai T. Tamaoka A. Homma M. (2017). Liquorice-induced Hypokalaemia in Patients Treated with Yokukansan Preparations: Identification of the Risk Factors in a Retrospective Cohort Study. BMJ Open 7 (6), e014218. 10.1136/bmjopen-2016-014218 PMC5623453 28619768

[B68] Shinno H. Inami Y. Inagaki T. Nakamura Y. Horiguchi J. (2008). Effect of Yi-Gan San on Psychiatric Symptoms and Sleep Structure at Patients with Behavioral and Psychological Symptoms of Dementia. Prog. Neuropsychopharmacol. Biol. Psychiatry 32 (3), 881–885. 10.1016/j.pnpbp.2007.12.027 18243460

[B69] Shinno H. Utani E. Okazaki S. Kawamukai T. Yasuda H. Inagaki T. (2007). Successful Treatment with Yi-Gan San for Psychosis and Sleep Disturbance in a Patient with Dementia with Lewy Bodies. Prog. Neuropsychopharmacol. Biol. Psychiatry 31 (7), 1543–1545. 10.1016/j.pnpbp.2007.07.002 17688986

[B70] Singh A. K. Rai S. N. Maurya A. Mishra G. Awasthi R. Shakya A. (2021). Therapeutic Potential of Phytoconstituents in Management of Alzheimer's Disease. Evid. Based Complement. Alternat Med. 2021, 5578574. 10.1155/2021/5578574 34211570 PMC8208882

[B71] Soraoka H. Oniki K. Matsuda K. Ono T. Taharazako K. Uchiyashiki Y. (2016). The Effect of Yokukansan, a Traditional Herbal Preparation Used for the Behavioral and Psychological Symptoms of Dementia, on the Drug-Metabolizing Enzyme Activities in Healthy Male Volunteers. Biol. Pharm. Bull. 39 (9), 1468–1474. 10.1248/bpb.b16-00248 27582327

[B72] Sterne J. A. Hernán M. A. Reeves B. C. Savović J. Berkman N. D. Viswanathan M. (2016). ROBINS-I: a Tool for Assessing Risk of Bias in Non-randomised Studies of Interventions. BMJ 355, i4919. 10.1136/bmj.i4919 27733354 PMC5062054

[B73] Sumiyoshi H. Mantani A. Nishiyama S. Fujiwaki S. Ohta S. Masuda Y. (2013). Yokukansan Treatment of Chronic Renal Failure Patients Receiving Hemodialysis, with Behavioral and Psychological Symptoms of Dementia: an Open-Label Study. Am. J. Geriatr. Psychiatry 21 (11), 1082–1085. 10.1016/j.jagp.2011.06.001 23567442

[B74] Sung W. S. Jeon S. R. Hong Y. J. Kim T. H. Shin S. Lee H. J. (2019). Efficacy, Safety, and Cost-Effectiveness Analysis of Adjuvant Herbal Medicine Treatment, Palmijihwang-Hwan, for Chronic Low Back Pain: a Study Protocol for Randomized, Controlled, Assessor-Blinded, Multicenter Clinical Trial. Trials 20 (1), 778. 10.1186/s13063-019-3776-7 31882016 PMC6935187

[B75] Takeyoshi K. Kurita M. Nishino S. Teranishi M. Numata Y. Sato T. (2016). Yokukansan Improves Behavioral and Psychological Symptoms of Dementia by Suppressing Dopaminergic Function. Neuropsychiatr. Dis. Treat. 12, 641–649. 10.2147/NDT.S99032 27042075 PMC4801203

[B76] Teranishi M. Kurita M. Nishino S. Takeyoshi K. Numata Y. Sato T. (2013). Efficacy and Tolerability of Risperidone, Yokukansan, and Fluvoxamine for the Treatment of Behavioral and Psychological Symptoms of Dementia: a Blinded, Randomized Trial. J. Clin. Psychopharmacol. 33 (5), 600–607. 10.1097/JCP.0b013e31829798d5 23948783

[B77] Terasawa K. Shimada Y. Kita T. Yamamoto T. Tosa H. Tanaka N. (1997). Choto-san in the Treatment of Vascular Dementia: a Double-Blind, Placebo-Controlled Study. Phytomedicine 4 (1), 15–22. 10.1016/s0944-7113(97)80022-0 23195240

[B78] Tewari D. Stankiewicz A. M. Mocan A. Sah A. N. Tzvetkov N. T. Huminiecki L. (2018). Ethnopharmacological Approaches for Dementia Therapy and Significance of Natural Products and Herbal Drugs. Front. Aging Neurosci. 10, 3. 10.3389/fnagi.2018.00003 29483867 PMC5816049

[B79] van der Linde R. M. Dening T. Stephan B. C. Prina A. M. Evans E. Brayne C. (2016). Longitudinal Course of Behavioural and Psychological Symptoms of Dementia: Systematic Review. Br. J. Psychiatry 209 (5), 366–377. 10.1192/bjp.bp.114.148403 27491532 PMC5100633

[B80] Ware J. E. Jr. Sherbourne C. D. (1992). The MOS 36-item Short-form Health Survey (SF-36). I. Conceptual Framework and Item Selection. Med. Care 30 (6), 473–483. 10.1097/00005650-199206000-00002 1593914

[B81] Wimo A. Guerchet M. Ali G. C. Wu Y. T. Prina A. M. Winblad B. (2017). The Worldwide Costs of Dementia 2015 and Comparisons with 2010. Alzheimers Dement 13 (1), 1–7. 10.1016/j.jalz.2016.07.150 27583652 PMC5232417

[B82] Xu B. Jiang X. Zhong Z. Chao W. (2007). Effect of Lemai Granules on 28 Cases of Vascular Dementia Accompanied by Issuing a Psychological Symptom. West China Pharm. J. 22 (5), 594. 10.13375/j.cnki.wcjps.2007.05.003

[B83] Xu M. (2018). Effects of Naoxintong Combined with Nimodipine on Vascular Dementia. Clin. J. Chin. Med. 10 (9), 40–42. 10.3969/j.issn.1674-7860.2018.09.018

[B84] Yang H. Wang H. Li X. Guo Z. (2012). Treatment of 60 Cases of Senile Dementia with Phlegm and Blood Stasis Obstruction Accompanied by Abnormal Mental Behavior. Shandong J. Traditional Chin. Med. 31 (8), 574–575. 10.16295/j.cnki.0257-358x.2012.08.002

[B85] Yao H. Gou Y. Zhou X. (2014). Therapeutic Analysis of Taohong Siwu Decoction Combined with Clopidogrel in the Treatment of Vascular Dementia. Guiding J. Traditional Chin. Med. Pharm. 20 (10), 59–60. 10.13862/j.cnki.cn43-1446/r.2014.10.020

[B86] Zhang J. Onakpoya I. J. Posadzki P. Eddouks M. (2015). The Safety of Herbal Medicine: from Prejudice to Evidence. Evid. Based Complement. Alternat Med. 2015, 316706. 10.1155/2015/316706 25838831 PMC4370194

[B87] Zhang Y. Lin C. Zhang L. Cui Y. Gu Y. Guo J. (2015). Cognitive Improvement during Treatment for Mild Alzheimer's Disease with a Chinese Herbal Formula: a Randomized Controlled Trial. PloS one 10 (6), e0130353. 10.1371/journal.pone.0130353 26076022 PMC4468068

[B88] Zhang Y. Ma C. Ge X. Wen Y. Yang X. Feng J. (2018). Clinical Study of Shugan Jieyu Capsule Combined with Buspirone Hydrochloride Tablets and Sertraline Hydrochloride Dispersible Tablets on Alzheimer's Disease with Depression and Anxiety Disorder. Hebei J. TCM 40 (8), 1166–1170. 10.3969/j.issn.1002-2619.2018.08.010

[B89] Zhang Z. (2012). Clinical Observation on the Treatment of 40 Cases of Senile Dementia with Mental and Behavioral Disorders. Forum Traditional Chin. Med. 27 (4), 28–29.

[B90] Zhou X. Li C. G. Chang D. Bensoussan A. (2019). Current Status and Major Challenges to the Safety and Efficacy Presented by Chinese Herbal Medicine. Medicines (Basel) 6 (1), 14. 10.3390/medicines6010014 PMC6473719 30669335

[B91] Zhou X. (2018). Observation on the Effect of Traditional Chinese Medicine in the Treatment of Patients with Senile Dementia. Home Med. (10), 22–23. 10.3969/j.issn.1671-4954.2018.10.024

[B92] Zhou Y. Wei D. (2015). Clinical Observation of Xiaoyao Powder in Treating Patients with Senile Dementia and Depression. J. Chin. Integr. Med. 7 (1), 25–26. 10.3969/j.issn.1674-4616.2015.01.008

[B93] Zhou Y. (2015). The Cinical Study of Xiaoyaosan Decoction Treatment of (Liver Depression and Spleen Deficiency) Depression in Alzheimer's Disease [Master's Degree]. Hubei: Hubei University of Chinese Medicine.

[B94] Zhu X. Hu J. Ding Y. Zhang T. (2019). Therapeutic Effect of the Method of Replenishing Qi, Removing Phlegm and Dredging Collaterals in Treating Vascular Dementia and its Influence on HCY Inflammatory Factors and Oxidative Stress Levels. Zhejiang Clin. Med. J. 21 (5), 643–645.

[B95] Zuo Q. (2017). Observation on the Curative Effect of Dihuangyinzi in Improving Alzheimer's Disease Complicated with Depression. Shanxi J. Traditional Chin. Med. 33 (8), 47.

