# Supplementary material for: Herbal Medicine for Behavioral and Psychological Symptoms of Dementia: A Systematic Review and Meta-Analysis
Source: Front Pharmacol. 2021 Jul 27;12:713287. doi: 10.3389/fphar.2021.713287 (PMC8353144; doi:10.3389/fphar.2021.713287)
Supplement: Supplementary file 1 [file DataSheet4.docx]

Supplement 4. Details of herbal medicine used

| **Study ID** | **Name** | **Dosage form** | **Main component (per day)** | **Additional component (per day)** |
| --- | --- | --- | --- | --- |
| Randomized controlled trial (36) | |  |  |  |
| Chen 1997 | Yizhi capsule | capsule | Panacis Quinquifolii Radix, Lycii Fructus, Curcumae Radix, Paeoniae Radix, Gastrodiae Rhizoma, Acori Graminei Rhizoma, etc | Not applicable |
| Chen 2013 | Naoling granule | granule | Chinese patent drugs | Not applicable |
| Chen 2020 | Yangxue Qingnao granule | granule | Chinese patent drugs | Not applicable |
| Du 2015 | Liuweidihuang pill | pill | Chinese patent drugs | Not applicable |
| Fang 2018 | Shugan Jieyu capsule | capsule | Chinese patent drugs | Not applicable |
| Furukawa 2017 | Yokukansan | powder | Atractylodis Rhizoma, Poria Sclerotium, Cnidii Rhizoma, Uncariae Ramulus cum Uncus, Angelicae Gigantis Radix, Bupleuri Radix, Glycyrrhizae Radix et Rhizoma | Not applicable |
| Gu 2018 | Liuweidihuang pill | pill | Chinese patent drugs | Not applicable |
| Guo 2011 | Zhibai Dihuang decoction | decoction | Anemarrhenae Rhizoma 9 g, Phellodendri Cortex 9 g, Rehmanniae Radix Preparata 24 g, Corni Fructus 12 g, Dioscoreae Rhizoma 12 g, Alismatis Rhizoma 9 g, Poria Sclerotium 9 g, Moutan Radicis Cortex 9 g | Not applicable |
| Han 2018 | Jianwei Yunao decoction | decoction | Codonopsis Pilosulae Radix 6 g, Atractylodis Rhizoma Alba 10 g, Pinelliae Tuber 10 g, Citri Unshius Pericarpium 15 g, Poria Sclertum Cum Pini Radix 10 g, Massa Medicata Fermentata 10 g, Zizyphi Semen 10 g, Rhodiolae Crenulate Radix 10 g, Gastrodiae Rhizoma 10 g, Glycyrrhizae Radix et Rhizoma 6 g | Not applicable |
| Hu 2015 | Bushen Tongluo decoction | decoction | Polygonati Rhizoma 30 g, Rehmanniae Radix Preparata 30 g, Polygoni Multiflori Radix 10 g, Epimedii Herba 15 g, Cuscutae Semen 20 g, Ginseng Radix 10 g, Salviae Miltiorrhizae Radix 15 g, Notoginseng Radix Et Rhizoma 3 g, Polygalae Radix 15 g, Cnidii Rhizoma 15 g, Citri Unshius Pericarpium 15 g, Ginkgonis Folium 15 g | -Kidney essence deficiency: Corni Fructus 10 g, Lycii Fructus 20 g Ligustri Fructus 20 g -Spleen qi deficiency: Astragali Radix 30 g, Atractylodis Rhizoma Alba 15 g, Poria Sclerotium 20 g -Qi and blood deficiency: Angelicae Gigantis Radix 10 g, Paeoniae Radix 10 g, Asini Corii Colla 10 g  -Phlegm turbidity: Acori Graminei Rhizoma 10 g, Pinelliae Tuber 10 g -yin deficiency with effulgent fire: Moutan Radicis Cortex 10 g, Fossilia Ossis Mastodi 30 g, Ostreae Testa 30 g |
| Huang 2019 | Bushen Jiannao decoction | decoction | Rehmanniae Radix Preparata 15 g, Corni Fructus 15 g, Polygoni Multiflori Radix 15 g, Gardeniae Fructus 15 g, Prunellae Spica 15 g, Carthami Flos 15 g, Lycii Fructus 15 g, Bupleuri Radix 15 g, Persicae Semen 15 g, Cistanchis Herba 15 g, Uncariae Ramulus cum Uncus 15 g, Paeoniae Radix 15 g, Testudinis Chinemis Plastrum et Carapax 15 g, Lilii Bulbus 15 g, Acori Graminei Rhizoma 15 g, Fossilia Ossis Mastodi 30 g, Ostreae Testa 30 g, Zizyphi Semen 30 g, Polygalae Radix 12 g, Curcumae Radix 12 g, Morindae Radix 10 g, Gastrodiae Rhizoma 10 g | Not applicable |
| Li 2018 | Bushen Yizhi decoction | decoction | Rehmanniae Radix Preparata 25 g, Lycii Fructus 15 g, Cnidii Rhizoma 12 g, Cnidi Fructus 15 g, Salviae Miltiorrhizae Radix 10 g, Polygoni Multiflori Radix 20 g, Acori Graminei Rhizoma 10 g, Ginseng Radix 12 g, Glycyrrhizae Radix et Rhizoma 10 g, Ligustri Fructus 10 g, Hirudo 10 g, Polygalae Radix 10 g | Not applicable |
| Li 2020 | Bugan Zhuangshen decoction | decoction | Corni Fructus 15 g, Polygoni Multiflori Radix 20 g, Rehmanniae Radix Preparata 30 g, Dioscoreae Rhizoma 30 g, Pelodiscis Carapax 20 g, Mori Fructus 30 g, Cuscutae Semen 15 g | -Flustered, insomnia, dizziness, white fur: Schisandrae Fructus 20 g, Longan Arillus 20 g, Atractylodis Rhizoma Alba 20 g, Poria Sclerotium 20 g, Polygalae Radix 20 g, Codonopsis Pilosulae Radix 30 g -Pale complexion, cold limbs, dizziness, lethatgy, dreaminess, much phlegm: Persicae Semen 15 g, Armeniacae Semen 1 g, Glycyrrhizae Radix et Rhizoma 10 g, Fritillariae Thunbergii Bulbus 20 g, Notoginseng Radix Et Rhizoma 20 g |
| Lin 2016 | Fufang Haishe capsule | capsule | Chinese patent drugs | Not applicable |
| Liu 2015 | Bushen Yizhi granule | granule | Rehmanniae Radix Preparata 10 g, Morindae Radix 10 g, Cistanchis Herba 10 g, Epimedii Herba 10 g, Dendrobii Caulis 10 g, Liriopis seu Ophiopogonis Tuber 10 g, Poria Sclerotium 10 g, Salviae Miltiorrhizae Radix 10 g, Lumbricus 10 g, Corni Fructus 6 g, Acori Graminei Rhizoma 6 g, Polygalae Radix 6 g, Schisandrae Fructus 6 g, Cnidii Rhizoma 6 g, Hirudo 3 g | Not applicable |
| Mizukami 2009 | Yokukansan | powder | Atractylodis Rhizoma, Poria Sclerotium, Cnidii Rhizoma, Uncariae Ramulus cum Uncus, Angelicae Gigantis Radix, Bupleuri Radix, Glycyrrhizae Radix et Rhizoma | Not applicable |
| Monji 2009 | Yokukansan | powder | Atractylodis Rhizoma, Poria Sclerotium, Cnidii Rhizoma, Uncariae Ramulus cum Uncus, Angelicae Gigantis Radix, Bupleuri Radix, Glycyrrhizae Radix et Rhizoma | Not applicable |
| Motohashi 2006 | Guanyuan granule | granule | Salviae Miltiorrhizae Radix, Carthami Flos, Cnidii Rhizoma, Paeoniae Radix, Cyperi Rhizoma, Aucklandiae Radix | Not applicable |
| Okahara 2010 | Yokukansan | powder | Atractylodis Rhizoma, Poria Sclerotium, Cnidii Rhizoma, Uncariae Ramulus cum Uncus, Angelicae Gigantis Radix, Bupleuri Radix, Glycyrrhizae Radix et Rhizoma | Not applicable |
| Pan 2014 | Sheng-Zhi-Ling oral liquid | oral liquid | Codonopsis Pilosulae Radix, Cinnamomi Ramulus, Paeoniae Radix Rubra, Glycyrrhizae Radix et Rhizoma, Poria Sclerotium, Zingiberis Rhizoma, Polygalae Radix, Acori Graminei Rhizoma, Fossilia Ossis Mastodi, Ostreae Testa | Not applicable |
| Pu 2014 | Modified Tongqiao Huoxue decoction | decoction | Persicae Semen 12 g, Carthami Flos 9 g, Paeoniae Radix Rubra 12 g, Cnidii Rhizoma 9 g, Agastachis Herba 0.5 g, Allii Fistulosi Bulbus 6 g, Zingiberis Rhizoma Recens 9 g, Zizyphi Fructus 7 pieces, Acori Graminei Rhizoma 10 g | Not applicable |
| Shen 2013 | Liuweidihuang pill | pill | Chinese patent drugs | Not applicable |
| Shen 2018 | Xiaoyao pill | pill | Chinese patent drugs | Not applicable |
| Shen 2019 | Xiaoyao pill | pill | Chinese patent drugs | Not applicable |
| Shi 2020 | Tianzhi granule | granule | Gastrodiae Rhizoma, Uncariae Ramulus cum Uncus, Nardotidis seu Sulculii Concha, Eucommiae Cortex, Loranthi Ramulus Et Folium, Caulis Polygoni Multiflori, Poria Sclertum Cum Pini Radix, Gardeniae Fructus, Sophorae Flos, Scutellariae Radix, Leonuri Herba, Achyranthis Radix | Not applicable |
| Teranishi 2013 | Yokukansan | powder | Atractylodis Rhizoma, Poria Sclerotium, Cnidii Rhizoma, Uncariae Ramulus cum Uncus, Angelicae Gigantis Radix, Bupleuri Radix, Glycyrrhizae Radix et Rhizoma | Not applicable |
| Terasawa 1997 | Choto-san | powder | Uncariae Ramulus cum Uncus, Citri Unshius Pericarpium, Pinelliae Tuber, Liriopis seu Ophiopogonis Tuber, Poria Sclerotium, Ginseng Radix, Chrysanthmi Flos, Saposhnikoviae Radix, Glycyrrhizae Radix et Rhizoma, Gypsum Fibrosum, Zingiberis Rhizoma Recens | Not applicable |
| Yao 2014 | Taohong Siwu decoction | decoction | Persicae Semen 15 g, Carthami Flos 10 g, Rehmanniae Radix Preparata 10 g, Angelicae Gigantis Radix 10 g, Carthami Flos 10 g, Paeoniae Radix 10 g | Not applicable |
| Zhang 2012 | Bunao mixture | decoction | Rehmanniae Radix Preparata 15 g, Cistanchis Herba 10 g, Cervi Cornu Colla 10 g, Lycii Fructus 15 g, Morindae Radix 10 g, Zizyphi Semen 15 g, Polygalae Radix 10 g, Acori Graminei Rhizoma 6 g, Schisandrae Fructus 6 g, Liriopis seu Ophiopogonis Tuber 10 g, Glycyrrhizae Radix et Rhizoma 6 g, Polygoni Multiflori Radix 15 g | Not applicable |
| Zhang 2015 | Yishen Huazhuo decoction | decoction | Epimedii Herba 10 g, Ligustri Fructus 10 g, Psoraleae Semen 10 g, Polygoni Multiflori Radix 10 g, Astragali Radix 10 g, Cnidii Rhizoma 6 g, Acori Graminei Rhizoma 6 g | Not applicable |
| Zhang 2018 | Liver Jieyu capsule | capsule | Chinese patent drugs | Not applicable |
| Zhou 2015a | Xiaoyaosan | decoction | Bupleuri Radix 15 g, Angelicae Gigantis Radix 15 g, Paeoniae Radix 15 g, Atractylodis Rhizoma Alba 15 g, Poria Sclerotium 15 g, Glycyrrhizae Radix et Rhizoma 6 g, Menthae Herba 6 g, Zingiberis Rhizoma Recens 6 g | Not applicable |
| Zhou 2015b | Xiaoyaosan | powder | Bupleuri Radix 15 g, Angelicae Gigantis Radix 15 g, Paeoniae Radix 15 g, Atractylodis Rhizoma Alba 15 g, Poria Sclerotium 15 g, Zingiberis Rhizoma Recens 15 g, Menthae Herba 6 g, Glycyrrhizae Radix et Rhizoma 6 g | Not applicable |
| Zhou 2018 | Qingnao decoction | decoction | Fossilia Ossis Mastodi 30 g, Codonopsis Pilosulae Radix 15 g, Polygalae Radix 15 g, Salviae Miltiorrhizae Radix 15 g, Alismatis Rhizoma 15 g, Curcumae Radix 15 g, Astragali Radix 15 g, Corni Fructus 15 g, Cannabis Semen 15 g, Arisaematis Rhizoma 15 g, Angelicae Gigantis Radix 15 g, Polygoni Multiflori Radix 15 g | Not applicable |
| Zhu 2019 | Buyang Huanwu decoction and Erchen decoction | decoction | Astragali Radix 30 g, Angelicae Gigantis Radix 10 g, Paeoniae Radix Rubra 10 g, Lumbricus 1 piece, Cnidii Rhizoma 10 g, Carthami Flos 3 g, Persicae Semen 15 g, Pinelliae Tuber 15 g, Citri Unshius Pericarpium 6 g, Poria Sclerotium 20 g, Glycyrrhizae Radix et Rhizoma 6 g | Not applicable |
| Zuo 2017 | Modified Dihuangyinzi | decoction | Rehmanniae Radix Preparata 20 g, Corni Fructus 20 g, Cistanchis Herba 20 g, Morindae Radix 20 g, Acori Graminei Rhizoma 15 g, Polygalae Radix 15 g, Poria Sclerotium 15 g, Albizziae Flos 15 g, Arisaematis Rhizoma 15 g, Dendrobii Caulis 10 g, Liriopis seu Ophiopogonis Tuber 10 g, Schisandrae Fructus 10 g, Zingiberis Rhizoma Recens 3 g, Menthae Herba 3 g, Zizyphi Fructus 2 pieces | Not applicable |
| Controlled clinical trial (2) | | | | |
| Kudoh 2016 | Ninjin’yoeito | powder | Rehmanniae Radix, Angelicae Gigantis Radix, Atractylodis Rhizoma Alba, Poria Sclerotium, Ginseng Radix, Cinnamomi Cortex, Polygalae Radix, Paeoniae Radix, Citri Unshius Pericarpium, Astragali Radix, Glycyrrhizae Radix et Rhizoma, Schisandrae Fructus | Not applicable |
| Xu 2018 | Naoxintong capsule | granule | Chinese patent drugs | Not applicable |
| Cohort (1) | | | | |
| Meguro 2018 | Yokukansankachimpihange | powder | Atractylodis Rhizoma, Poria Sclerotium, Cnidii Rhizoma, Uncariae Ramulus cum Uncus, Angelicae Gigantis Radix, Bupleuri Radix, Glycyrrhizae Radix et Rhizoma, Citri Unshius Pericarpium, Pinelliae Tuber | Not applicable |
| Before-after study (12) | | | | |
| Hayashi 2010 | Yokukansan | powder | Atractylodis Rhizoma, Poria Sclerotium, Cnidii Rhizoma, Uncariae Ramulus cum Uncus, Angelicae Gigantis Radix, Bupleuri Radix, Glycyrrhizae Radix et Rhizoma | Not applicable |
| Guo 2011 | Zhibai Dihuang decoction | decoction | Anemarrhenae Rhizoma 9 g, Phellodendri Cortex 9 g, Rehmanniae Radix Preparata 24 g, Evodiae Fructus 12 g, Dioscoreae Rhizoma 12 g, Alismatis Rhizoma 9 g, Poria Sclerotium 9 g, Moutan Radicis Cortex 9 g | Not applicable |
| Yang 2012 | Zhibai Dihuang decoction | decoction | Persicae Semen 12 g, Carthami Flos 9 g, Paeoniae Radix Rubra 12 g, Cnidii Rhizoma 9 g, Agastachis Herba 0.5 g, Arisaematis Rhizoma 12 g, Acori Graminei Rhizoma 12 g, Pinelliae Tuber 12 g, Atractylodis Rhizoma Alba 12 g, Citri Unshius Pericarpium 9 g | Not applicable |
| Ohsawa 2017 | Ninjin’yoeito | powder | Rehmanniae Radix, Angelicae Gigantis Radix, Atractylodis Rhizoma Alba, Poria Sclerotium, Ginseng Radix, Cinnamomi Cortex, Polygalae Radix, Paeoniae Radix, Citri Unshius Pericarpium, Astragali Radix, Glycyrrhizae Radix et Rhizoma, Schisandrae Fructus | Not applicable |
| Iwasaki 2005 | Yokukansan | powder | Atractylodis Rhizoma, Poria Sclerotium, Cnidii Rhizoma, Uncariae Ramulus cum Uncus, Angelicae Gigantis Radix, Bupleuri Radix, Glycyrrhizae Radix et Rhizoma | Not applicable |
| Iwasaki 2012 | Yokukansan | powder | Atractylodis Rhizoma, Poria Sclerotium, Cnidii Rhizoma, Uncariae Ramulus cum Uncus, Angelicae Gigantis Radix, Bupleuri Radix, Glycyrrhizae Radix et Rhizoma | Not applicable |
| Manabe 2020 | Yokukansankachimpihange | powder | Atractylodis Rhizoma, Poria Sclerotium, Cnidii Rhizoma, Uncariae Ramulus cum Uncus, Angelicae Gigantis Radix, Bupleuri Radix, Glycyrrhizae Radix et Rhizoma, Citri Unshius Pericarpium, Pinelliae Tuber | Not applicable |
| Shinno 2008 | Yokukansan | NR | Atractylodis Rhizoma, Poria Sclerotium, Cnidii Rhizoma, Uncariae Ramulus cum Uncus, Angelicae Gigantis Radix, Bupleuri Radix, Glycyrrhizae Radix et Rhizoma | Not applicable |
| Sumiyoshi 2013 | Yokukansan | powder | Atractylodis Rhizoma, Poria Sclerotium, Cnidii Rhizoma, Uncariae Ramulus cum Uncus, Angelicae Gigantis Radix, Bupleuri Radix, Glycyrrhizae Radix et Rhizoma | Not applicable |
| Kawanabe 2010 | Yokukansan | powder | Atractylodis Rhizoma, Poria Sclerotium, Cnidii Rhizoma, Uncariae Ramulus cum Uncus, Angelicae Gigantis Radix, Bupleuri Radix, Glycyrrhizae Radix et Rhizoma | Not applicable |
| Xu 2007 | Lemai Granule | granule | Chinese patent drugs | Not applicable |
| Nagata 2012 | Yokukansan | powder | Atractylodis Rhizoma, Poria Sclerotium, Cnidii Rhizoma, Uncariae Ramulus cum Uncus, Angelicae Gigantis Radix, Bupleuri Radix, Glycyrrhizae Radix et Rhizoma | Not applicable |
| Case series/case report (1) | | | | |
| Shinno 2007 | Yokukansan | powder | Atractylodis Rhizoma, Poria Sclerotium, Cnidii Rhizoma, Uncariae Ramulus cum Uncus, Angelicae Gigantis Radix, Bupleuri Radix, Glycyrrhizae Radix et Rhizoma | Not applicable |
