# Supplementary material for: Herbal Medicine for Behavioral and Psychological Symptoms of Dementia: A Systematic Review and Meta-Analysis
Source: Front Pharmacol. 2021 Jul 27;12:713287. doi: 10.3389/fphar.2021.713287 (PMC8353144; doi:10.3389/fphar.2021.713287)
Supplement: Supplementary file 2 [file DataSheet6.docx]

**Supplement 6. Details of adverse events reported**

| **Study ID** | **Adverse event** |
| --- | --- |
| Randomized controlled trial (36) | |
| Chen 1997 | None |
| Chen 2013 | (A) 2 (insomnia), 1 (constipation) (B) 3 (drowsiness), 6 (insomnia), 4 (headache), 15 (dry mouth), 1 (constipation), 4 (extrapyramidal symptom) |
| Chen 2020 | Not reported |
| Du 2015 | (A) 2 (drowsiness), 1 (headache, dizziness), 1 (insomnia), 3 (extrapyramidal symptom) (B) 4 (drowsiness), 4 (headache, dizziness), 3 (insomnia), 5 (extrapyramidal symptom) |
| Fang 2018 | Not reported |
| Furukawa 2017 | (A) 4 (hypokalemia), 2 (acute heart failure and cholelithiasis; not related to HM) (B) 1 (anorexia) |
| Gu 2018 | (A) 1 (headache, dizziness), 2 (insomnia), 1 (extrapyramidal symptom) (B) 5 (narcolepsy), 4 (headache, dizziness), 2 (insomnia), 3 (extrapyramidal symptom) |
| Guo 2011 | (A) 1 (nausea), 1 (diarrhea) (B) 1 (nausea) |
| Han 2018 | Not reported |
| Hu 2015 | Not reported |
| Huang 2019 | (A) 4 (fatigue), 6 (insomnia), 1 (drowsiness), 1 (liver dysfunction) (B) 3 (fatigue), 5 (insomnia), 2 (drowsiness), 1 (liver dysfunction) |
| Li 2018 | (A) 4 (nausea), 2 (dizziness), 4 (narcolepsy), 3 (dry mouth), 2 (weight gain), 2 (constipation) (B) 5 (anorexia), 3 (dizziness), 4 (narcolepsy), 2 (dry mouth), 3 (weight gain), 2 (constipation) |
| Li 2020 | Not reported |
| Lin 2016 | (A) 2 (nausea), 3 (dry mouth), 2 (constipation) (B) 6 (dry mouth), 4 (drowsiness), 3 (nausea), 2 (dizziness), 1 (insomnia) |
| Liu 2015 | Not reported |
| Mizukami 2009 | (A) 3 (vomiting/diarrhea, nausea, epigastric distress), 1 (hypokalemia and sedation), 1 (hypokalemia), 1 (leg edema) |
| Monji 2009 | (A) 2 (hypokalemia), 1 (extrapyramidal sign) (B) none |
| Motohashi 2006 | None |
| Okahara 2010 | None |
| Pan 2014 | None |
| Pu 2014 | (A) 2 (over-sedation), 2 (dizziness), 1 (cognitive decline), 3 (constipation) (B) 10 (over-sedation), 4 (dizziness), 8 (cognitive decline), 1 (constipation) |
| Shen 2013 | (A) 2 (drowsiness), 1 (headache, dizziness), 1 (insomnia), 2 (extrapyramidal symptom) (B) 4 (drowsiness), 5 (headache, dizziness), 3 (insomnia), 5 (extrapyramidal symptom) |
| Shen 2018 | (A) 1 (nausea, vomiting), 2 (dry mouth), 1 (headache) (B) 5 (nausea, vomiting), 5 (dry mouth), 3 (headache) |
| Shen 2019 | (A) 1 (skin itching), 1 (nausea, vomiting), 1 (dizziness), 1 (headache) (B) 1 (fatigue), 3 (dizziness), 2 (poor appetite), 4 (diarrhea), 3 (headache), 3 (sleep disturbance) |
| Shi 2020 | (A) 2 (urinary tract infection), 1 (abnormal renal function), 1 (bloating) (B1) 1 (diarrhea), 1 (arrhythmia), 1 (loss of appetite) (B2) 1 (insomnia) |
| Teranishi 2013 | (A) 1 (fracture), 1 (head injury), 1 (fall with contusion), 15 (constipation), 1 (fatigue), 2 (insomnia) (B1) 1 (fall with contusion), 1 (over-sedation), 1 (swallowing difficulty), 1 (stridor), 1 (sudden death), 22 (constipation), 5 (muscle rigidity), 3 (sialorrhea), 3 (sedation), 2 (fatigue), 1 (insomnia) (B2) 1 (hallucination and delusion), 1 (refusal to eat), 1 (fall with contusion), 20 (constipation), 1 (muscle rigidity), 1 (sialorrhea), 1 (sedation), 1 (fatigue) |
| Terasawa 1997 | (A) 5 (urticaria, diarrhea, appetite loss, heartburn, hypertension) (B) 2 (oral bitterness, liver dysfunction) |
| Yao 2014 | Not reported |
| Zhang 2012 | (A) 0 (B) 31 (myotonia), 25 (tremor and akathisia,), 12 (torsion spasticity), 2 (tardive dyskinesia), etc. |
| Zhang 2015 | (A) 1 (cerebral infarction), 1 (catching cold), 1(abnormal LFT), 1 (arthralgia), 1 (constipation) (B) 2 (diarrhea), 1 (cerebral infarction), 1 (catching cold), 1 (abnormal LFT), 1 (insomnia) |
| Zhang 2018 | Not reported |
| Zhou 2015a | (A) 0 (B) 2 (constipation, insomnia) |
| Zhou 2015b | Not reported |
| Zhou 2018 | Not reported |
| Zhu 2019 | (A) 2 (diarrhea) (B) 1 (sleep disturbance) |
| Zuo 2017 | Not reported |
| Controlled clinical trial (2) | |
| Kudoh 2016 | Not reported |
| Xu 2018 | Not reported |
| Cohort (1) | |
| Meguro 2018 | Not reported |
| Before-after study (12) | |
| Hayashi 2010 | 1 (hypokalemia), 1 (tendency toward somnolence), 1 (leg edema), 1 (over-sedated and gait disorder) |
| Guo 2011 | None |
| Yang 2012 | 1 (anxiety), 1 (dizziness), 2 (anorexia), 2 (flustered), 1 (insomnia), 1 (diarrhea) |
| Ohsawa 2017 | 1 (deteriorated consciousness levels with hypotension), 1 (worsened backache), 1 (fracture from falling), 1 (γ-GTP elevation), 1 (vomiting) |
| Iwasaki 2005 | 1 (drowsiness) |
| Iwasaki 2012 | 4 (hypokalaemia), 1 (spasticity and worsening of BPSD), 1 (worsening hypotension), etc (edema, gastrointestinal dysfunction, spasticity, and exacerbation of delusions and hallucinations) |
| Manabe 2020 | None |
| Shinno 2008 | None |
| Sumiyoshi 2013 | 1 (nausea), 1 (tiredness) |
| Kawanabe 2010 | 1 (sialorrhea) |
| Xu 2007 | Not reported |
| Nagata 2012 | None |
| Case report (1) | |
| Shinno 2007 | None |
