# Supplementary material for: Herbal Medicine for Behavioral and Psychological Symptoms of Dementia: A Systematic Review and Meta-Analysis
Source: Front Pharmacol. 2021 Jul 27;12:713287. doi: 10.3389/fphar.2021.713287 (PMC8353144; doi:10.3389/fphar.2021.713287)
Supplement: Supplementary file 3 [file DataSheet3.docx]

Supplement 3. Basic characteristics of other included studies

| Study ID | Sample size (included →analyzed) | Mean age (year) | | Sex (M:F) | | Population (Diagnosis) | | Pattern identification | | (A) Treatment intervention | | Treatment duration / Follow-up | Outcome | |
| --- | --- | --- | --- | --- | --- | --- | --- | --- | --- | --- | --- | --- | --- | --- |
|  |  |  |  |  |  |  |  |  |  | (B) Control intervention | | Results | | |
| Controlled clinical trial (2) | | | | | | | | | | | | | | |
| Kudoh 2016 | 23(12:11)  →23(12:11) | (A) 74.5 ± 5.4 (B) 74.9 ± 3.6 | | | (A) 12(4:8) (B) 11(4:7) | | -AD (DSM-IV, NINCDS-ADRDA) -MMSE 15-23 (A) 20.5 ± 1.9 (B) 20.3 ± 2.5 -ADAS-cog (A) 36.1 ± 4.5 (B) 35.6 ± 4.3 -NPI (A) 33.6 ± 4.7 (B) 33.9 ± 4.7 | | NA | | HM + (B) | 2 year / NR | | 1. MMSE 2. ADAS-cog 3. NPI |
|  |  |  |  |  |  |  |  |  |  |  |  | 1. no comparison between the groups (no raw data presented)  2. TG<CG (p<0.01) (no raw data presented)  3. no comparison between the groups (33.7±4.6 12 / 35.2±4.3 11)  (1) Delusions: no comparison between the groups (4.8±1.2 12 / 5.0±1.3 11)  (2) Hallucinations: no comparison between the groups (3.1±1.0 12 / 3.2±1.3 11)  (3) Agitation: no comparison between the groups (2.8±0.8 12 / 2.7±0.8 11)  (4) Depression: TG<CG (p<0.01) (5.7±1.3 12 / 7.1±0.7 11)  (5) Anxiety: no comparison between the groups (5.2±1.0 12 / 6.0±1.3 11)  (6) Elation: TG>CG (p<0.05) (2.8±0.8 12 / 1.8±0.6 11)  (7) Indifference: no comparison between the groups (1.8±0.6 12 / 1.8±0.6 11)  (8) Disinhibition: no comparison between the groups (1.8±0.6 12 / 1.8±0.6 11)  (9) Irritability: no comparison between the groups (3.2±0.8 12 / 3.1±1.3 11)  (10) Aberrant motor behavior: no comparison between the groups (2.8±0.6 12 / 2.6±0.8 11) | | |
|  |  |  |  |  |  |  |  |  |  |  | Donepezil 5mg/day |  |  |  |
| Xu 2018 | 60(30:30)  →60(30:30) | (A) 64.85 ± 0.35 (B) 64.95 ± 0.25 | | | (A) 30(17:13) (B) 30(18:12) | | -VD (ICD-10, DSM-IV) | | NA | | HM + (B) | 1 month / NR | | 1. TER (unclear) 2. Cognitive function  3. ADL  4. BPSD |
|  |  |  |  |  |  |  |  |  |  |  | Nimodipine 90-360/day | 1. TG>CG (p<0.01) (29/30, 22/30)  2. TG>CG (p<0.01) (81.8±9.5 30 / 64.8±10.1 30)  3. TG>CG (p<0.05) (93.2±2.7 30 / 86.1±2.6 30)  4. TG>CG (p<0.05) (96.3±5.6 30 / 77.6±5.3 30) | | |
| Cohort (1) | | | | | | | | | | | | | | |
| Meguro 2018 | 46(23:23)  →46(23:23) | | (A) 77.7 ± 5.6 (B) 78.0 ± 6.7 | | (A) 23(4:19) (B) 23(5:18) | | -AD (NINCDS-ADRDA) -MMSE≥10 (A) 17.80 ± 4.6 (B) 17.7 ± 4.7 -BEHAVE-AD (A) 13.2 ± 21.0 (B) 10.3 ± 10.1 | | NA | | HM + (B) | 2 week / NR | | 1. MMSE 2. Digit Symbol 3. BEHAVE-AD-FW  *raw data was not presented |
|  |  |  |  |  |  |  |  |  |  |  | Donepezil 10mg/day | 1. no significant difference (16.3±3.8 23 / 18.3±4.8 23)  2. no significant difference (18.1±9.7 23 / 19.4±7.6 23)  3. (1) Paranoid and Delusional Ideation: no significant difference (p=0.206)  (2) Hallucinations: no significant difference (p=0.285)  (3) Activity Disturbances: no significant difference (p=0.152)  (4) Aggressiveness: no significant difference (p=0.471)  (5) Diurnal Rhythm Disturbances: TG<CG* (p=0.018)  (6) Affective Disturbance: no significant difference (p=0.089)  (7) Anxiety and Phobias: no significant difference (p=0.060) | | |
| Before-after study (12) | | | | | | | | | | | | | | |
| Hayashi 2010 | 29→26 | | 74.8 ± 9.2 | | 15:11 | | -AD (ICD-10, DSM-IV, NINCDS-ADRDA)  -MMSE  11.9 ± 8.6  -NPI (at least one symptom score of four or more in the NPI subscales)  26.8 ± 11.5  -HIS≤6 | | NA | | HM | 4 week / NR | | 1. NPI  2. MMSE  3. DAD  4. Zarit burden interview  5. SDS  6. Serum potassium |
|  |  |  |  |  |  |  |  |  |  |  |  | 1. 26.8±11.5 to 17.2±12.9 (p=0.0009)  (1) Delusions: 2.2±3.6 to 1.4±2.9 (p=0.2617)  (2) Hallucinations: 1.1±2.3 to 0.3±0.9 (p=0.0313)  (3) Agitation/aggression: 3.9±4.0 to 2.1±2.6 (p=0.0416)  (4) Dysphoria: 0.8±1.4 to 0.4±0.9 (p=0.2188)  (5) Anxiety: 2.5±3.7 to 1.3±2.8 (p=0.0195)  (6) Euphoria: 0.5±1.5 to 0.6±1.8 (p=1.0000)  (7) Apathy: 6.0±5.1 to 4.7±4.8 (p=0.0503)  (8) Disinhibition: 2.2±3.4 to 1.4±2.2 (p=0.1250)  (9) Irritability/lability: 3.2±3.3 to 1.6±2.8 (p=0.0161)  (10) Aberrant motor activity: 4.5±4.4 to 3.4±4.0 (p=0.0459)  2. 11.9±8.6 to 11.3±8.6 (p=0.9761)  3. 29.6±34.0 to 35.8±36.0 (p=0.1210)  4. 32.5±21.9 to 31.3±27.1 (p=0.8750)  5. 44.0±7.9 to 43.6±10.1 (p=0.5923)  6. 4.1±0.4 to 4.0±0.5 (p=0.0406) | | |
| Guo 2011 | 30→30 | | 75.6 ± 5.02 | | 17:13 | | -AD (NINCDS-ADRDA) -MMSE 5-12 7.4 ± 1.99 -BEHAVE-AD≥8 -HIS≤4 | | liver-kidney deficiency | | HM | 1 month / NR | | 1. TER (BEHAVE-AD) 2. BEHAVE-AD 3. MMSE |
|  |  |  |  |  |  |  |  |  |  |  |  | 1. 17/30  2. 13.5±2.79 to 9.8±3.69 (p<0.01)  3. 7.4±1.99 to 8.9±2.68 (p<0.01) | | |
| Yang 2012 | 60→60 | | 74.9 ± 4.02 | | 35:25 | | -AD (NINCDS-ADRDA) -MMSE 5-12 -BEHAVE-AD≥8 13.6 ± 2.67 -HIS≤4 | | phlegm and stasis obstruction | | HM | 4 week / NR | | 1. BEHAVE-AD 2. Severe impairment battery 3. ADL |
|  |  |  |  |  |  |  |  |  |  |  |  | 1. 13.6±2.67 to 8.6±3.56 (p<0.01)  2. 61.2±3.57 to 63.7±4.23 (p=0.017)  3. 30.3±2.78 to 29.3±2.73 (p=0.018) | | |
| Ohsawa 2017 | 20→17 | | 82.6 ± 7.7 | | 4:16 | | -AD (NINCDS-ADRDA) -Anorexia/apathy (NPI subcategory scores for anorexia and apathy>3) -MMSE≤26 17.32 ± 1.29 -NPI 13.75 ± 1.54 | | NA | | HM | 12 week / NR | | 1. NPI 2. Vitality index 3. MMSE |
|  |  |  |  |  |  |  |  |  |  |  |  | 1. 13.75±1.54 to 5.88±0.96 (p<0.001)  (1) Delusions: 0.20±0.16 to 0.18±0.10 (p>0.05)  (2) Hallucinations: 0.10±0.07 to 0.00±0.00 (p>0.05)  (3) Agitation/aggression: 0.35±0.17 to 0.29±0.17 (p>0.05)  (4) Dysphoria: 0.80±0.49 to 0.06±0.06 (p>0.05)  (5) Anxiety: 0.30±0.16 to 0.24±0.24 (p>0.05)  (6) Euphoria: 0.00±0.00 to 0.00±0.00 (p>0.05)  (7) Apathy: 5.85±0.65 to 3.31±0.51 (p<0.001)  (8) Disinhibition: 0.10±0.10 to 0.12±0.12 (p>0.05)  (9) Irritability/lability: 0.10±0.07 to 0.00±0.00 (p>0.05)  (10) Aberrant motor activity: 0.45±0.25 to 0.44±0.27 (p>0.01)  (11) Night-time Disturbance: 0.45±0.22 to 0.25±0.19 (p>0.05)  (12) Eating Disturbance: 5.05±0.60 to 0.94±0.43 (p<0.001)  (13) Distress Total: 5.35±0.91 to 2.56±0.57 (p<0.001)  2. 7.05±0.43 to 7.94±0.39 (p<0.05)  3. 17.32±1.29 to 19.44±1.30 (p<0.001) | | |
| Iwasaki 2005 | 14→14 | | 73.3 | | 9:5 | | -DLB (Consensus guidelines for the clinical and pathologic diagnosis of DLB) -Visual hallucinations -MMSE  17.5 ± 6.8 -NPI  34.7 ± 21.8 | | NA | | HM | 4 week / NR | | 1. NPI 2. Barthel Index 3. MMSE |
|  |  |  |  |  |  |  |  |  |  |  |  | 1. 34.7±21.8 to 13.5±14.5 (p=0.0008)  (1) hallucination: 7.5±3.3 to 1.5±1.8 (p<0.0001)  2. 76.6±28.3 to 82.2±24.4 (p=0.0027)  3. 17.5±6.8 to 18.6±7.9 (p>0.05) | | |
| Iwasaki 2012 | 60→54 | | 78.2 ± 5.8 | | 30:33 | | -DLB (Consensus guidelines for the clinical and pathologic diagnosis of DLB) -MMSE 18.0 ± 7.0 -NPI (NPI score≥4 on at least one of the subscales) 30.5 ± 18.5 | | NA | | HM | 4 week / NR | | 1. NPI 2. MMSE 3. DAD 4. Zarit burden interview 5. Serum potassium |
|  |  |  |  |  |  |  |  |  |  |  |  | 1. 30.5±18.5 to 16.9±16.2 (p=0.0000)  (1) Delusions: 7.5±3.3 to 4.4±3.8 (p=0.0000)  (2) Hallucinations: 8.8±3.5 to 3.9±3.5 (p=0.0000)  (3) Agitation/aggression: 5.4±3.5 to 3.0±2.7 (p=0.0056)  (4) Dysphoria: 3.8±2.6 to 1.5±1.5 (p=0.0000)  (5) Anxiety: 4.3±3.4 to 2.6±3.1 (p=0.0009)  (6) Euphoria: 2.8±2.4 to 1.1±1.1 (p=0.1250)  (7) Apathy: 5.3±2.9 to 3.8±3.3 (p=0.0241)  (8) Disinhibition: 4.7±3.1 to 2.4±3.1 (p=0.0313)  (9) Irritability/lability: 4.9±3.6 to 1.6±2.3 (p=0.0005)  (10) Aberrant motor activity: 5.4±3.5 to 3.5±3.9 (p=0.0166)  2. 18.0±7.0 to 19.2±7.4 (p=0.0019)  3. 56.0±31.6 to 57.4±32.6 (p=0.5080)  4. 33.1±17.9 to 29.8±18.7 (p=0.0244)  5. 4.2±0.4 to 3.9±0.5 (p=0.0084) | | |
| Manabe 2020 | 13→11 | | 76.5 ± 2.3 | | 8:3 | | -DLB (Fourth consensus report of the DLB Consortium) -MMSE 25.3 ± 4.5 -MoCA 20.6 ± 5.2 -RBD (RBDSQ≥5, PSG) | | NA | | HM | 4 week / NR | | 1. NPI (night-time behavior disturbance) 2. VAS (frequency) 3. VAS (severity) 4. MDS-UPDRS III 5. Serum potassium |
|  |  |  |  |  |  |  |  |  |  |  |  | 1. 5.9±2.1 to 2.5±1.8 (p<0.01)  2. 2.6±0.7 to 1.4±0.9 (p<0.01)  3. 1.6±0.5 to 0.9±0.5 (p<0.05)  4. 2.1±3.2 to 2.1±3.2 (p>0.05)  5. 4.5±0.4 to 4.4±0.4 (p=0.260) | | |
| Shinno 2008 | 10→5 | | 81.6 ± 6.9 | | 1:4 | | -AD (DSM-IV, NINCDS-ADRDA) or DLB (Consensus guidelines for the clinical and pathologic diagnosis of DLS) -MMSE<24  18.4 ± 4.5 -NPI-NH 34.0 ± 6.5 | | NA | | HM | 4 week / NR | | 1. NPI-NH 2. PSG 3. PSQI |
|  |  |  |  |  |  |  |  |  |  |  |  | 1. 34.0±6.5 to 12.8±6.6 (p<0.01)  (1) Delusions: 7.4±2.5 to 1.8±2.4 (p<0.01)  (2) Hallucinations: 4.6±3.1 to 1.6±2.5 (p<0.05)  (3) Agitation/aggression: 3.0±2.4 to 1.2±1.6 (p<0.05)  (4) Dysphoria: 2.0±1.9 to 0.6±0.5 (p>0.05)  (5) Anxiety: 3.4±2.8 to 1.4±1.7 (p<0.05)  (6) Euphoria: 0.2±0.4 to 0±0 (p>0.05)  (7) Apathy: 4.0±1.2 to 2.8±1.3 (p>0.05)  (8) Disinhibition: 2.0±1.9 to 0.4±0.9 (p>0.05)  (9) Irritability/lability: 3.8±1.5 to 1.2±1.1 (p>0.05)  (10) Aberrant motor activity: 3.6±1.7 to 1.8±1.3 (p>0.05)  2.  (1) Total sleep time (min): 255.4±85.4 to 352.6±122.5 (p<0.01)  (2) Sleep efficiency (%): 46.4±8.8 to 66.2±8.3 (p<0.01)  (3) Stage 1, % of TST: 55.8±13.8 to 34.1±15.6 (p>0.05)  (4) Stage 2, % of Total sleep time: 34.8±9.4 to 50.0±15.5 (p<0.01)  (5) Stage 3 + 4, % of Total sleep time: 1.10±2.1 to 1.51±1.4 (p>0.05)  (6) Stage REM, % of Total sleep time: 8.31±7.3 to 14.4±7.7 (p>0.05)  (7) No. of awakenings, >20s: 74.8±34.8 to 38.8±3.4 (p<0.05)  (8) No. of awakenings, >1min: 25.6±8.8 to 11.0±2.0 (p<0.05)  (9) Per sleep time (hour) index, no. per hour: 39.0±19.4 to 18.2±6.2 (p<0.05)  3. 10.2±2.2 to 6.6±1.1 (p<0.05) | | |
| Sumiyoshi 2013 | 12→11 | | 68.0 ± 7.3 | | 7:4 | | -AD or VD (DSM-IV) -MMSE<20 10.5 ± 6.0 -NPI 25.3 ± 17.6 | | NA | | HM | 4 week / NR | | 1. NPI 2. Barthel index 3. Serum K+ |
|  |  |  |  |  |  |  |  |  |  |  |  | 1. 25.3±17.6 to 8.36±4.46 (p=0.0069)  2. 47.6±21.2 to 50.4±22.1 (p=0.1441)  3. 3.89±0.56 to 3.78±0.46 (p=0.6465) | | |
| Kawanabe 2010 | 7→7 | | 76.2 ± 5.4 | | 5:2 | | -PDD (DSM-IV, Consensus guidelines for the clinical and pathologic diagnosis of DLS) -MMSE 21.4 ± 4.0 | | NA | | HM | 4 week / 4 week | | 1. NPI 2. NPI subscale score for hallucinations 3. MMSE 4. Barthel index 5. UPDRS-III |
|  |  |  |  |  |  |  |  |  |  |  |  | 1. 12.4±7.4 to 6.4±4.2 (p=0.018)  2. 5.4±3.6 to 2.8±4.1 (p=0.027)  3. 21.4±4.0 to 22.5±4.5 (p=0.039)  4. raw-data was not presented. (p>0.05)  5. raw-data was not presented. (p>0.05) | | |
| Xu 2007 | 28→28 | | 80 ± 10.66 | | 9:19 | | -VD (DSM-IV) -BEHAVE-AD 10.92 ± 6.24 | | NA | | HM | 60-90 day / NR | | 1. TER (cognitive symptom, ADL) 2. HDS 3. BEHAVE-AD 4. GBS 5. DAD |
|  |  |  |  |  |  |  |  |  |  |  |  | 1. 4.23±5.35 to 6.00±6.16 (p<0.05)  2. 10.92±6.24 to 9.15±5.87 (p<0.01)  3. 83.04±22.17 to 79.58±24.04 (p<0.01)  4. 3.96±5.83 to 4.65±5.84 (p>0.05) | | |
| Nagata 2012 | 13→12 | | 71.2 ± 6.5 | | 9:4 | | -VD (NINDS-AIREN) -MMSE 15.3 ± 8.5 -NPI 33.0 ± 17.3 | | NA | | HM | 4 week / NR | | 1. MMSE 2. NPI 3. Barthel index 4. DAD 5. UPDRS |
|  |  |  |  |  |  |  |  |  |  |  |  | 1. 15.3±8.5 to 15.8±9.0 (p>0.05)  2. 33.0±17.3 to 23.6±13.9 (p<0.05)  (1) Agitation: 6.9±4.2 to 4.1±3.7 (p<0.05)  (2) Disinhibition: 5.1±2.2 to 3.3±2.2 (p<0.05)  3. 362±31.9 to 40.8±30.3 (p>0.05)  4. 20.5±18.9 to 24.6±20.8 (p>0.05)  5. 17.2±11.0 to 16.1±10.9 (p>0.05) | | |
| Case report (1) | | | | | | | | | | | | | | |
| Shinno 2007 | 1 | | 81 | | Male | | -DLB (neuroimmaging) -Psychosis and sleep disturbance (PSG) -MMSE: 20 -NPI: 37 | | NA | | HM | 4 week / NR | | 1. MMSE 2. NPI 3. PSG |
|  |  |  |  |  |  |  |  |  |  |  |  | 1. 20 to 21  2. 37 to 9  (1) Delusions: 8 to 1  (2) Hallucinations: 3 to 0  (3) Agitation/aggression: 4 to 1  (4) Dysphoria: 4 to 1  (5) Anxiety: 6 to 2  (6) Euphoria: 0 to 0  (7) Apathy: 3 to 2  (8) Disinhibition: 1 to 0  (9) Irritability/lability: 6 to 1  (10) Aberrant motor activity: 2 to 1  3.  (1) total sleep time (min): 352 to 504  (2) sleep efficiency (%): 48 to 79  (3) N of awakenings > 20s: 111 to 35  (4) N of awakenings > 1min: 37 to 10  (5) Stage I (min): 163 to 188  (6) Stage II (min): 125 to 235  (7) Stage III+IV (min): 2.3 to 9  (8) Stage REM (min): 44 to 72  (9) periodic limb movement during sleep index (N/h): 70.1 to 28.3 | | |

AD, Alzheimer's disease; ADAS-cog, Alzheimer's disease assessment scale–cognitive subscale; ADL, activities of daily living; BEHAVE-AD, behavioral pathology in Alzheimer's disease rating scale; BEHAVE-AD-FW, behavioral pathology in Alzheimer's disease frequency weighted severity scale; BPSD, behavioral and psychological symptoms of dementia; CG, control group; DAD, disability assessment of dementia; DLB, dementia with Lewy bodies; DSM, diagnostic and statistical manual of mental disorders; GBS, Gottfries-Bråne-Steen; HDS, Hasegawa's dementia scale; HIS, Hachinski ischemia score; HM, herbal medicine; ICD, the international statistical classification of diseases and related health problems; MDS-UPDRS, the movement disorder society-sponsored revision of the unified Parkinson's disease rating scale; MMSE, mini-mental state examination; MoCA, Montreal cognitive assessment; NA, not applicable; NINCDS-ADRDA, national institute of neurological and communicative diseases and stroke/Alzheimer's disease and related disorders association; NINDS-AIREN, national Institute of neurological disorders and stroke and association internationale pour la Recherché et l'Enseignement en neurosciences; NPI, neuropsychiatric inventory; NPI-NH, neuropsychiatric inventory-nursing home; NR, not recorded; PDD, Parkinson’s disease dementia; PSG, polysomnography; PSQI, Pittsburgh sleep quality index; RBD, REM sleep behavior disorder; RBDSQ, REM sleep behavior disorder screening questionnaire; REM, rapid eye movement; SDS, self-rating depression scale; TER, total effective rate; TG, treatment group; UPDRS, the unified Parkinson's disease rating scale; VAS, visual analogue scale; VD, vascular dementia.
