# Supplementary material for: Herbal Medicine for Behavioral and Psychological Symptoms of Dementia: A Systematic Review and Meta-Analysis
Source: Front Pharmacol. 2021 Jul 27;12:713287. doi: 10.3389/fphar.2021.713287 (PMC8353144; doi:10.3389/fphar.2021.713287)
Supplement: Supplementary file 4 [file DataSheet5.docx]

**Supplement 5**. **Details of risk of bias in the included studies**

1. Controlled clinical trial/Cohort study

|  | 1. Selection of participants | 2. Confounding variables | 3. Measurement of intervention (exposure) | 4. Blinding of outcome assessment | 5. Incomplete outcome data | 6. Selective outcome reporting |
| --- | --- | --- | --- | --- | --- | --- |
| Kudoh 2016 | Low | Low | Low | Low | Low | Low |
| Xu 2018 | Unclear | Low | Unclear | Unclear | Low | Unclear |
| Meguro 2018 | Low | Low | Low | Low | Low | Low |

1. Before-after studies with no control group

|  | 1. Was the study question or objective clearly stated? | 2. Were eligibility/selection criteria for the study population prespecified and clearly described? | 3. Were the participants in the study representative of those who would be eligible for the test/service/intervention in the general or clinical population of interest? | 4. Were all eligible participants that met the prespecified entry criteria enrolled? | 5. Was the sample size sufficiently large to provide confidence in the findings? | 6. Was the test/service/intervention clearly described and delivered consistently across the study population? | 7. Were the outcome measures prespecified, clearly defined, valid, reliable, and assessed consistently across all study participants? | 8. Were the people assessing the outcomes blinded to the participants' exposures/interventions? | 9. Was the loss to follow-up after baseline 20% or less? Were those lost to follow-up accounted for in the analysis? | 10. Did the statistical methods examine changes in outcome measures from before to after the intervention? Were statistical tests done that provided p values for the pre-to-post changes? | 11. Were outcome measures of interest taken multiple times before the intervention and multiple times after the intervention (i.e., did they use an interrupted time-series design)? | 12. If the intervention was conducted at a group level (e.g., a whole hospital, a community, etc.) did the statistical analysis take into account the use of individual-level data to determine effects at the group level? |
| --- | --- | --- | --- | --- | --- | --- | --- | --- | --- | --- | --- | --- |
| Hayashi 2010 | Yes | Yes | Yes | Cannot determine | No | Yes | Yes | Not reported | Yes | Yes | No | No |
| Guo 2011 | Yes | Yes | Yes | Cannot determine | No | Yes | Yes | Not reported | Yes | Yes | No | No |
| Yang 2012 | Yes | Yes | Yes | Cannot determine | Yes | Yes | Yes | Not reported | Yes | Yes | No | No |
| Ohsawa 2017 | Yes | Yes | Yes | Cannot determine | No | Yes | Yes | Not reported | Yes | Yes | Yes | No |
| Iwasaki 2005 | Yes | Yes | Yes | Cannot determine | No | Yes | Yes | Not reported | Yes | Yes | No | Yes |
| Iwasaki 2012 | Yes | Yes | Yes | Cannot determine | Yes | Yes | Yes | Not reported | Yes | Yes | Yes | No |
| Manabe 2020 | Yes | Yes | Yes | Yes | No | Yes | Yes | Not reported | Yes | Yes | No | Yes |
| Shinno 2008 | Yes | Yes | Yes | Yes | No | No | Yes | Not reported | No | Yes | No | No |
| Sumiyoshi 2013 | Yes | Yes | Yes | Yes | No | No | Yes | Not reported | Yes | Yes | Yes | No |
| Kawanabe 2010 | Yes | No | Yes | Not applicable | No | Yes | Yes | Not reported | Yes | Yes | Yes | No |
| Xu 2007 | Yes | No | Yes | Not applicable | No | Yes | Yes | Not reported | Yes | Yes | No | No |
| Nagata 2012 | Yes | Yes | Yes | Yes | No | Yes | Yes | Not reported | Yes | Yes | No | No |

1. Case report

|  | 1. Was the study question or objective clearly stated? | 2. Was the study population clearly and fully described, including a case definition? | 3. Were the cases consecutive? | 4. Were the subjects comparable? | 5. Was the intervention clearly described? | 6. Were the outcome measures clearly defined, valid, reliable, and implemented consistently across all study participants? | 7. Was the length of follow-up adequate? | 8. Were the statistical methods well-described? | 9. Were the results well-described? |
| --- | --- | --- | --- | --- | --- | --- | --- | --- | --- |
| Shinno 2007 | Yes | Yes | Not applicable | Not applicable | Yes | Yes | No | Not applicable | Yes |
