# Supplementary material for: Herbal Medicine for Behavioral and Psychological Symptoms of Dementia: A Systematic Review and Meta-Analysis
Source: Front Pharmacol. 2021 Jul 27;12:713287. doi: 10.3389/fphar.2021.713287 (PMC8353144; doi:10.3389/fphar.2021.713287)
Supplement: Supplementary file 5 [file DataSheet2.docx]

**Supplement 2. Excluded studies after full-text review**

1) not original article: 1

1. Takeyoshi K, Kurita M, Nishino S, et al. Yokukansan improves behavioral and psychological symptoms of dementia by suppressing dopaminergic function. *Neuropsychiatr Dis Treat.* 2016;12:641-649.

2) not clinical study: 6

1. 施慧芬. 顺时调节阴阳治疗梗塞性痴呆睡眠时相紊乱. 2006年神经科学新进展国际研讨会; 2006; 北京.

2. 金若米. 老年性痴呆用养心汤. *开卷有益（求医问药）.* 2007(8):41.

3. 周磊, 杨文明. 老年性痴呆的中医药研究. *中医药临床杂志.* 2007;19(2):183-185.

4. Mizukami K. Kampo therapy as an alternative to pharmacotherapy using antipsychotic medicines for behavioral and psychological symptoms of dementia (BPSD). *Psychogeriatrics.* 2008;8(3):137-141.

5. Zhihong L, Jie G, Jin X, Tingyan M, Jie D, Tiwari JK. Traditional Chinese Medicine for Senile Dementia. *Evidence-based Complementary & Alternative Medicine (eCAM).* 2012;2012:1-13.

6. Tohda C, Watari H. [Kamikihito improves memory dysfunction in Alzheimer's disease]. *Nihon Yakurigaku Zasshi.* 2015;145(5):224-228.

3) only abstract available without raw data: 11

1. 李初霞, 李茂霞. 利脑心胶囊治疗脑血管性痴呆的疗效观察. 第六次全国中西医结合血瘀证及活血化瘀研究学术大会论文汇编; 2005; 中国吉林延吉.

2. Wiebrecht A. A randomised, observer-blind, controlled trial of the traditional Chinese medicine Yi-Gan San for improvement of behavioral and psychological symptoms and activities of daily living in dementia patients: commentary. *Deutsche zeitschrift fur akupunktur.* 2007;50(3):48‐49.

3. Furuhashi Y, Shin K, Oshima H, Furuhashi S. Comparative efficacy of risperidone versus Yi-Gan san on behavioural and psychological symptoms of dementia. *Alzheimer's and Dementia.* 2009;5(4):235.

4. Shinno H. Herbal medicine for psychiatric symptoms and sleep disturbances in patients with dementia. *International Journal of Neuropsychopharmacology.* 2010;13:40-41.

5. Ishida Y, Hayashi Y, Tsuchiya T. Usefulness of treatment with yokukansan, a traditional Japanese medicine, in patients with dementia with Lewy bodies: Retrospective observational study for efficacy and safety of long-term treatment. *Journal of Neurology.* 2013;260:S166.

6. Matsuda Y, Kishi T, Shibayama H, Iwata N. Yokukansan in the treatment of behavioural and psychological symptoms of dementia. *European Neuropsychopharmacology.* 2013;23:S541.

7. 张伟. 六味地黄丸和奥氮平对老年痴呆精神行为症状改善作用的比较分析. 2014中华医学会第十二次全国精神医学学术会议论文集; 2014; 西安.

8. 王猛, 刘帅, 柳净, 李晶, 纪勇. 百乐眠胶囊治疗阿尔茨海默病伴精神行为症状的疗效观察. 中华医学会第十八次全国神经病学学术会议论文集; 2015; 成都.

9. Pin Tan H, Hsiao KY, Ouyang WC. Traditional chinese medicine improves circadian rhythm disturbance of an elder with dementia: A case report. *International Psychogeriatrics.* 2019;31:118.

10. Tian J, Shi J, Ni J, et al. THE EFFECTIVENESS OF STEP REGIMEN FOR ALZHEIMER'S DISEASE IN COGNITION AND BEHAVIOR: A REAL-WORLD STUDY. *Alzheimer's and Dementia.* 2019;15(7):P583-P584.

11. Urabe S, Tsuchida H, Tamura J, et al. THE EFFECTS OF ORENGEDOKUTO FOR THE IMPROVEMENT OF AGGRESSION FOR PATIENTS WITH DEMENTIA. *Alzheimer's and Dementia.* 2019;15(7):P931-P932.

4) not about dementia: 2

1. Zhao H. Observation on Treatment of Senile Depressive Dementia with Traditional Chinese Medicine. *China Health Industry.* 2014(1):187,189.

2. Soraoka H, Oniki K, Matsuda K, et al. The effect of Yokukansan, a traditional herbal preparation used for the behavioral and psychological symptoms of dementia, on the drug-metabolizing enzyme activities in healthy male volunteers. *Biological and Pharmaceutical Bulletin.* 2016;39(9):1468-1474.

5) accompany diseases other than dementia: 2

1. Machida A, Yamada Y, Kimura S, Kozaki K, Toba K. The effect of YGS (Yi-Gan-San) on BPSD and care burden of dementia. *Japanese Journal of Geriatrics.* 2010;47(3):262-263.

2. Matsuoka T, Narumoto J, Shibata K, et al. Effect of Toki-Shakuyaku-San on Regional Cerebral Blood Flow in Patients with Mild Cognitive Impairment and Alzheimer's Disease. *Evidence-based Complementary & Alternative Medicine (eCAM).* 2012;2012:1-5.

6) not report diagnostic criteria of dementia: 8

1. Kimura T, Hayashida H, Furukawa H, Miyauchi D, Takamatsu J. Five cases of frontotemporal dementia with behavioral symptoms improved by Yokukansan. *Psychogeriatrics.* 2009;9(1):38-43.

2. Utumi Y, Iseki E, Murayama N, et al. Effect of Rikkunshi-to on appetite loss found in elderly dementia patients: A preliminary study. *Psychogeriatrics.* 2011;11(1):34-39.

3. Duan F, Lu X. Comparison of the effects of Liuwei Dihuang Wan and Olanzapine on improving the mental and behavioral symptoms of senile dementia. *Journal of China Prescription Drug.* 2014;12(10):58-59.

4. Fu Y. A comparative study on the effect of Liuwei Dihuang Wan and Olanzapine on improving the mental and behavioral symptoms of senile dementia. *Modern Medicine Journal of China.* 2015;17(7):55-56.

5. Tan B. Clinical Study on Treatment of Senile Dementia with Depression by Tianzhi Granule. *Asia-Pacific Traditional Medicine.* 2016;12(11):129-130.

6. Xu K. Longshoushenge pill in the treatment of frontotemporal dementia: a case report. *The Journal of Medical Theory and Practice.* 2016;29(11):1493.

7. Ji A. Observation on the curative effect of Ditan Decoction combined with donepezil in the treatment of Alzheimer's disease with abnormal mental behavior. *Home Medicine.* 2017(11):159-160.

8. Liang S, Liao Q. Analysis of the effect of Zhibai Dihuang Decoction combined with donepezil in the treatment of patients with senile dementia with abnormal mental behavior. *Contemporary Medicine Forum.* 2020;18(8):213-214.

7) not about oral HM: 3

1. 孙波. *老年期抑郁症与轻度阿尔茨海默病患者治疗前后血清BDNF水平的变化及APOE基因多态性研究* [硕士], 苏州大学; 2010.

2. Ahn HI, Hyun MK. Effectiveness of integrative medicine program for dementia prevention on cognitive function and depression of elderly in a public health center. *Integrative Medicine Research.* 2019;8(2):133-137.

3. Feng H, Cui X, Deng L. Study on Sleep Quality of Alzheimer's Disease with Self-made Anshen Powder Ironing Umbilical Therapy. *Medical Innovation of China.* 2020;17(25):125-128.

8) not report details of HM: 6

1. Shi H. Adjust of Yin-Yang Punctually Inordinated Dormancy Time for the Vascular Dementia. *Chinese Journal Of integrative Medicine On Cardio-/Cerebrovascuiar Disease.* 2007;5(1):20-22.

2. Guo Z, Chen X, Xing B, Zhang J. Clinical study of reinhartdt and sea cucumber capsule combined with donepezil in treatment of psychological and behavioral symptoms in patients with Alzheimers disease. *Zhejiang Medical Journal.* 2013;35(23):2089-2092.

3. Chen Y. *Research of Clinical Curative Effect and Relevant Mechanism in Modified Yam Pill in The Treatment of Vascular Dementia* [Master's degree], Hubei University of Chinese Medicine; 2015.

4. Yu E, Liao Z, Tan Y, et al. Efficacy and tolerability of memantine combined with Fufang Haishe Capsules in the treatment of moderate to severe agitation symptoms of Alzheimer's disease. *Chinese Medical Journal.* 2017;97(27):2091-2094.

5. Chen Y, Yu E, Liao Z, Tan Y, Qiu Y, Zhu J. Clinical study of memantine hydrochloride combined with Fufang Haishe Capsule in the treatment of Alzheimer's disease. *Zhejiang Journal of Clinical Medicine.* 2018;20(4):598-599,602.

6. Zhang R, Yuan Q, Wu Y, Liang S, Xu L, Wang M. Analysis of Curative Effect of Pengshi Guntan Pills in Treating Mental and Behavioral Symptoms of Alzheimer's Disease with Phlegm-heat Blocking Orifices. *Zhejiang Journal of Clinical Medicine.* 2020;22(5):680-682.

9) comparison of different HMs: 2

1. 张凤华, 张根芳. 神衰宁丸与奥氮平治疗老年痴呆精神行为症状的疗效比较. *中国伤残医学.* 2015(4):121-122.

2. 曹泽慧. 分析比较六味地黄丸和奥氮平对老年痴呆精神行为 症状的改善作用. *医药前沿.* 2018;8(4):320.

10) using other EATM other than HM: 3

1. Yuan S. Observation of curative effectiveness of vascular dementia treated with electronic acupuncture and herb broth. *Journal of clinical acupuncture and moxibustion.* 2002;18(8):40.

2. Chen LP, Wang FW, Zuo F, Jia JJ, Jiao WG. Clinical research on comprehensive treatment of senile vascular dementia. *Journal of traditional chinese medicine = chung i tsa chih ying wen pan.* 2011;31(3):178‐181.

3. 张玉涛, 赵赞, 冯永, 袁晓亮, 刘建. 补肾益智活血汤联合穴位贴敷佐治阿尔茨海默病临床评价. *中国药业.* 2020;29(22):60-62.

11) not report outcome of interest: 37

1. Cai Y, Du Ky Xie JG. Senile dementia of Alzheimer type treated by traditional Chinese medicine. *Chinese journal of gerontology.* 1994.

2. Chou LW, Yang JF, Liu YH. Treatment of vascular dementia in 72 cases with Jianshen Yangnao Decoction combined with brain active element. *Henan journal of traditional chinese medicine and pharmacy.* 1995;10(3):36‐37.

3. Xu J. Experience in treating senile dementia according to differentiation of syndromes. *Journal of Traditional Chinese Medicine.* 1996;16(3):176-181.

4. 林水淼, 杨柏灿. 养心健脑液治疗Alzheimer痴呆的临床研究. *上海中医药大学上海市中医药研究院学院.* 1996(Z1):44-47.

5. Huang D. TCM treatment of senile dementia - a report of 2 cases. *Journal of Traditional Chinese Medicine.* 1997;17(2):103-105.

6. Du G, Chen K, Zhou W, et al. Clinical effect of tianma-cuzhi granules on senile vascular dementia. *Zhongguo Zhong yao za zhi = Zhongguo zhongyao zazhi = China journal of Chinese materia medica.* 1998;23(11):695-698, inside back cover.

7. Hou AL, Wang L, Pu Y. Clinical observation of treatment of multi-infarctional dementia by herbs, oxygen and acupuncture. *Shanghai journal of acupuncture and moxibustion.* 1998;17(2):12‐13.

8. 付存穰. 中药为主治疗老年人多发梗塞性痴呆28例. *河南中医药学刊.* 1999;14(3):13-14.

9. 张继全, 刘桂芹, 李英. 复聪除愚汤治疗外伤性痴呆84例临床观察. *北京中医.* 1999(01):3-5.

10. Zheng L, Zhuang LX, Li YH. Observations on the curative effect of acupuncture and herbs on vascular dementia. *Shanghai journal of acupuncture and moxibustion.* 2000;19(3):8‐10.

11. Wang Y, Ma X. Clinical Observation on Treatment of 21 Cases of Senile Dementia with Yishen Huayu Jiangzhuo Decoction. *Modern Journal of Integrated Traditional Chinese and Western Medicine.* 2001;10(3):222-223.

12. Zhang Q. Three Cases of Senile Vascular Dementia Treated with the Method of Nourishing the Kidney, Nourishing Heart and Removing Blood Stasis. *Journal of Traditional Chinese Medicine.* 2001;42(6):336-337.

13. Li ZR, Mu YY, Ouyang Q. Clinic control research of Alzheimer's disease by the combination of acupuncture and Danggui Shaoyao San (DGSYS) of TCM. *Chinese Journal of Clinical Rehabilitation.* 2002;6(19):2848-2849.

14. Liu C, Zhou L, Shui Z. Tongqiao huoxue tang and buyang huanwu tang for treatment of vascular dementia - a report of 36 cases. *Journal of Traditional Chinese Medicine.* 2003;23(4):243-245.

15. Fan XZ, Yang BL. Clinical study of shuizhitong capsule in treating senile vascular dementia. *Zhongguo zhong xi yi jie he za zhi zhongguo zhongxiyi jiehe zazhi = chinese journal of integrated traditional and western medicine.* 2004;24(8):694‐697.

16. Liao XL, Chen KY, Zheng ZR, Liu H, Zheng KY. Clinical study of Shoulingjiannao capsule in improvement of brain function for patients with vascular dementia. *Chinese journal of clinical rehabilitation.* 2004;8(1):112‐114.

17. Liu Y, Wei H, Shan B, Liu Q. Clinical observation on effect of dishengzhu decoction in treating vascular dementia. *Zhongguo zhong xi yi jie he za zhi [chinese journal of integrated traditional and western medicine].* 2004;24(9):837‐838.

18. Suzuki T, Futami S, Igari Y, et al. A Chinese herbal medicine, choto-san, improves cognitive function and activities of daily living of patients with dementia: a double-blind, randomized, placebo-controlled study. *Journal - American Geriatrics Society.* 2005;53(12):2238-2240.

19. Lin SM, Wang J, Zhou RQ, Yu ZH. Clinical effect in treatment of Alzheimer disease based on the conditions of heart and kidney. *Chinese journal of clinical rehabilitation.* 2006;10(11):162‐164.

20. Maruyama M, Tomita N, Iwasaki K, et al. Benefits of combining donepezil plus traditional Japanese herbal medicine on cognition and brain perfusion in Alzheimer's disease: A 12-week observer-blind, donepezil monotherapy controlled trial [15]. *Journal of the American Geriatrics Society.* 2006;54(5):869-871.

21. Xu Q. Treatment of 36 Cases of Senile Dementia with Bushen Xingshen Decoction. Proceedings of the 3rd Zhejiang Midwest Science and Technology Forum (Volume 6 Chinese and Western Medicine Sub-volume); 2006; Quzhou, Zhejiang, China.

22. Zhu AH, Tian JZ, Zhong J, Yang CZ, Shi J, Yin JX. A clinical study on a randomized, double-blind control of Chinese medicine granules in treatment of vascular dementia. *Zhongguo Zhong yao za zhi [China journal of Chinese materia medica].* 2006;31(20):1722‐1725.

23. 田波. 补肾活血化浊汤治疗老年性痴呆18例疗效观察. *山西中医学院学报.* 2006;7(3):43-44.

24. He H. The application of Traditional Chinese Medicine (TCM) in the treatment of vascular dementia. *Chinese journal of information on traditional chinese medicine.* 2007;14(2):60‐61.

25. Higashi K, Rakugi H, Yu H, Moriguchi A, Shintani T, Ogihara T. Effect of kihito extract granules on cognitive function in patients with Alzheimer's-type dementia. *Geriatrics & Gerontology International.* 2007;7(3):245-251.

26. Iwasaki K, Kato S, Monma Y, et al. A pilot study of banxia houpu tang, a traditional Chinese medicine, for reducing pneumonia risk in older adults with dementia. *Journal of the american geriatrics society.* 2007;55(12):2035‐2040.

27. 梁秋花, 付存穰. 老年人多发梗死性痴呆的中医治疗与护理. *中外医疗.* 2008;27(24):85-85.

28. 李刚. 中医辨证治疗多发梗塞性痴呆体会. *内蒙古中医药.* 2010;29(2):7-7.

29. 陈文武, 黄一苇, 方建. 美金刚联合天智颗粒治疗老年性痴呆的临床观察. 世界中联第三届中医、中西医结合老年医学学术大会论文集; 2010; 河南南阳.

30. 侯延喜. 中西医结合治疗脑梗死后并发痴呆70例疗效观察. *河北医科大学学报.* 2011;32(8):934-936.

31. Li J. Analysis of the effect of Tongqiao Huoxue Decoction in the treatment of insomnia and senile dementia. *Guide of Chinese Medicine.* 2012;10(17):606-607.

32. 王少华, 石凌燕. 天智颗粒联合安理申治疗血管性痴呆疗效观察. *中国实用神经疾病杂志.* 2012;15(11):57-59.

33. 马洁琼. 通窍活血汤治疗失眠及老年性痴呆临床观察. *河南中医.* 2013;33(11):1947-1948.

34. 侯荷叶. 中西药结合治疗失眠、阿尔茨海默病的临床观察. *临床合理用药杂志.* 2013;6(25):49-50.

35. 张文华, 沈红梁. 通窍活血汤加减治疗老年性痴呆精神行为症状临床研究. *亚太传统医药.* 2017;13(18):155-156.

36. Yu S, Zhu Y, Wang M, Xu J. Clinical Observation of Fufang Haishe Capsule in the Treatment of Alzheimer's Disease and Early-onset Dementia Mental and Behavioral Symptoms. *Zhejiang Journal of Integrated Traditional Chinese and Western Medicine.* 2018;28(3):197-200.

37. Watari H, Shimada Y, Matsui M, Tohda C. Kihito, a traditional Japanese kampo medicine, improves cognitive function in Alzheimer's disease patients. *Evid Based Complement Alternat Med.* 2019;2019.

12) use duplicate data: 8

1. Motohashi K, Shi Z, Sei Y. Clinical Study of Guanyuan Granules on Behavior-Psychological Symptoms and Cognitive Function of Vascular Dementia. Proceedings of the Sixth National Conference of Integrated Traditional Chinese and Western Medicine on Blood Stasis Syndrome and Activating Blood to Remove Stasis; 2005; Yanji, Jilin.

2. Machida A, Yamada Y, Kimura S, Kanzaki K, Toba K. Effect of long-term administration of yokukansan on peripheral symptoms of dementia and feeling of burden of long-term care. *Japanese Journal of Geriatrics.* 2010;47(3):262-263.

3. Guo Z, Chen X, Xing B, Luo S, Shen Y. Clinical Observation of Zhibai Dihuang Decoction Combined with Donepezil in the Treatment of Senile Dementia with Mental and Behavioral Abnormalities. The 2011 Zhejiang Medical Association Psychiatry Branch Academic Conference Papers of the Geriatric Mental Disorders Group; 2011; Longquan, Zhejiang, China.

4. Iwasaki K, Kosaka K, Mori H, et al. OPEN LABEL TRIAL TO EVALUATE THE EFFICACY AND SAFETY OF YOKUKANSAN, A TRADITIONAL ASIAN MEDICINE, IN DEMENTIA WITH LEWY BODIES. *Journal of the American Geriatrics Society.* 2011;59(5):936-938.

5. Sumiyoshi H, Mantani A, Nishiyama S, et al. Yokukansan treatment in chronic renal failure patients with dementia receiving hemodialysis: an open label study. *The American journal of geriatric psychiatry : official journal of the American Association for Geriatric Psychiatry.* 2011;19(10):906-907.

6. Zhang Z. Clinical Study on Treatment of 40 Cases of Senile Dementia with Mental and Behavioral Disorders. Proceedings of the 11th Annual Academic Conference of the Mental Disease Professional Committee of the Chinese Integrative Medicine Association; 2012; Yiwu.

7. Pu Z, Fei Y, Lin Y, Xia J. Comparison of the curative effect of oxcarbazepine and Tongqiaohuoxue decoction in the treatment of agitation in patients with blood stasis and internal obstruction vascular dementia. Paper presented at: 2014 Academic Annual Meeting of Psychiatry Branch of Zhejiang Medical Association, Seventh Annual Meeting of Psychiatrist Branch of Zhejiang Medical Association2014; Huzhou, Zhejiang, China.

8. Shi J, Tian J, Wei M, et al. EFFICACY AND SAFETY OF TIANZHI GRANULE IN MILD TO MODERATE VASCULAR DEMENTIA: a MULTICENTRE, RANDOMIZED, DOUBLE-BLIND, THREE-ARMS TRIAL. *Alzheimer's & dementia.* 2018;14(7):P295‐P296.

13) not available full-text: 4

1. Wu X, Jin L. Clinical Research on Vascular Dementia Treated with Integrating Therapy of Traditional Chinese Medicine and Western Medicine. *Chinese journal of the practical chinese with modern medicine.* 2003.

2. Iwasaki K, Satoh-Nakagawa T, Maruyama M, et al. A randomized, observer-blind, controlled trial of the traditional Chinese medicine Yi-Gan San for improvement of behavioral and psychological symptoms and activities of daily living in dementia patients. *Journal of Clinical Psychiatry.* 2005;66(2):248-252.

3. 毕敏. *复智散治疗阿尔茨海默病的临床及PET研究* [博士], 哈尔滨医科大学; 2011.

4. Dang Gui Shao Yao San reduces dementia symptoms. *Journal of Chinese Medicine.* 2020(122):74-74.
