# Supplementary material for: Herbal Medicine for Behavioral and Psychological Symptoms of Dementia: A Systematic Review and Meta-Analysis
Source: Front Pharmacol. 2021 Jul 27;12:713287. doi: 10.3389/fphar.2021.713287 (PMC8353144; doi:10.3389/fphar.2021.713287)
Supplement: Supplementary file 6 [file DataSheet1.docx]

**Supplement 1. Search terms used in each database**

**MEDLINE via PubMed**

|  | Searches | Results |
| --- | --- | --- |
| #1 | Dementia[MeSH] OR dement*[Title/Abstract] OR Alzheimer*[Title/Abstract] OR “Lewy body”[Title/Abstract] OR Huntington*[Title/Abstract] OR Parkinson*[Title/Abstract] OR “Pick disease”[Title/Abstract] OR “cognitive impairment”[Title/Abstract] | 399,150 |
| #2 | “Plants, Medicinal”[MeSH] OR “Drugs, Chinese Herbal”[MeSH] OR “Medicine, Chinese Traditional”[MeSH] OR “Medicine, Kampo”[MeSH] OR “Medicine, Korean Traditional”[MeSH] OR “Herbal Medicine”[MeSH] OR “Prescription Drugs”[MeSH] OR “traditional Korean medicine”[Title/abstract] OR “traditional Chinese medicine”[Title/abstract] OR “traditional oriental medicine”[Title/abstract] OR “Kampo medicine”[Title/abstract] OR herb*[Title/abstract] OR decoction*[Title/abstract] OR botanic*[Title/abstract] | 226,010 |
| #3 | #1 AND #2 | **3,776** |

**EMBASE via Elsevier**

|  | Searches | Results |
| --- | --- | --- |
| #1 | 'dementia'/exp OR 'dement*':ab,ti OR ‘Alzheimer disease’/exp OR ‘Alzheimer*’:ab,ti OR ‘Lewy body’/exp OR ‘Lewy body’:ab,ti OR ‘Huntington*’:ab,ti OR ‘Parkinson disease’/exp OR ‘Parkinson’:ab,ti OR ‘Pick disease’:ab,ti OR ‘cognitive impairment’:ab,ti | 633,149 |
| #2 | ‘medicinal plant’/exp OR ‘medicinal plant’:ab,ti OR ‘herbaceous agent’/exp OR ‘herbaceous agent’:ab,ti OR ‘chinese medicine’/exp OR ‘chinese medicine’:ab,ti OR ‘kampo medicine’/exp OR ‘kampo medicine’:ab,ti OR ‘kampo medicine (drug)’/exp OR ‘kampo medicine (drug)’:ab,ti OR ‘korean medicine’/exp OR ‘korean medicine’:ab,ti OR ‘herbal medicine’/exp OR ‘herbal medicine’:ab,ti OR ‘prescription drug’/exp OR ‘prescription drug’:ab,ti OR ‘oriental medicine’/exp OR ‘oriental medicine’:ab,ti OR ‘herb’/exp OR ‘herb’:ab,ti OR ‘decoction’:ab,ti OR ‘botanic’:ab,ti | 386,116 |
| #3 | #1 AND #2 | **7,743** |

**CENTRAL**

|  | Searches | Results |
| --- | --- | --- |
| #1 | MeSH descriptor: [Dementia] explode all trees | 5,964 |
| #2 | (dement* OR Alzheimer* OR “Lewy body” OR Huntington* OR Parkinson* OR “Pick disease” OR “cognitive impairment”):ti,ab,kw | 34,558 |
| #3 | MeSH descriptor: [Plants, Medicinal] explode all trees | 940 |
| #4 | MeSH descriptor: [Drugs, Chinese Herbal] explode all trees | 3,576 |
| #5 | MeSH descriptor: [Medicine, Chinese Traditional] explode all trees | 1,179 |
| #6 | MeSH descriptor: [Medicine, Kampo] explode all trees | 46 |
| #7 | MeSH descriptor: [Medicine, Korean Traditional] explode all trees | 32 |
| #8 | MeSH descriptor: [Herbal Medicine] explode all trees | 62 |
| #9 | MeSH descriptor: [Prescription Drugs] explode all trees | 107 |
| #10 | (“traditional Korean medicine” OR “traditional Chinese medicine” OR “traditional oriental medicine” OR “Kampo medicine” OR herb* OR decoction* OR botanic*):ti,ab,kw | 18,194 |
| #13 | ((#1 OR #2) AND (#3 OR #4 OR #4 OR #6 OR #7 OR #8 OR #9 OR #10)) in Trials | **419** |

**AMED via EBSCO**

|  | Searches | Results |
| --- | --- | --- |
| #1 | Dementia[SU] OR dement*[TX] OR Alzheimer*[TX] OR “Lewy body”[TX] OR Huntington*[TX] OR Parkinson*[TX] OR “Pick disease”[TX] OR “cognitive impairment”[TX] | 6,788 |
| #2 | “Plants, Medicinal”[SU] OR “Drugs, Chinese Herbal”[SU] OR “Medicine, Chinese Traditional”[SU] OR “Medicine, Kampo”[SU] OR “Medicine, Korean Traditional”[SU] OR “Herbal Medicine”[SU] OR “Prescription Drugs”[SU] OR “traditional Korean medicine”[TX] OR “traditional Chinese medicine”[TX] OR “traditional oriental medicine”[TX] OR “Kampo medicine”[TX] OR herb*[TX] OR decoction*[TX] OR botanic*[TX] | 33,818 |
| #3 | #1 AND #2 | **471** |

**CINAHL via EBSCO**

|  | Searches | Results |
| --- | --- | --- |
| #1 | Dementia[MH] OR dement*[TX] OR Alzheimer*[TX] OR “Lewy body”[TX] OR Huntington*[TX] OR Parkinson*[TX] OR “Pick disease”[TX] OR “cognitive impairment”[TX] | 220,660 |
| #2 | “Plants, Medicinal”[MH] OR “Drugs, Chinese Herbal”[MH] OR “Medicine, Chinese Traditional”[MH] OR “Medicine, Kampo”[MH] OR “Medicine, Korean Traditional”[MH] OR “Herbal Medicine”[MH] OR “Prescription Drugs”[MH] OR “traditional Korean medicine”[TX] OR “traditional Chinese medicine”[TX] OR “traditional oriental medicine”[TX] OR “Kampo medicine”[TX] OR herb*[TX] OR decoction*[TX] OR botanic*[TX] | 116,064 |
| #3 | #1 AND #2 | **7,945** |

**PsycARTICLES via ProQuest**

|  | Searches | Results |
| --- | --- | --- |
| #1 | SU(Dementia) OR ‘dement*’ OR ‘Alzheimer*’ OR ‘Lewy body’ OR ‘Huntington*’ OR ‘Parkinson*’ OR ‘Pick disease’ OR ‘cognitive impairment’ | 28,534 |
| #2 | SU(Plants, Medicinal) OR SU(Drugs, Chinese Herbal) OR SU(Medicine, Chinese Traditional) OR SU(Medicine, Kampo) OR SU(Medicine, Korean Traditional) OR SU(Herbal Medicine) OR SU(Prescription Drugs) OR AB('traditional Korean medicine') OR AB('traditional Chinese medicine') OR AB('traditional oriental medicine') OR AB('Kampo medicine') OR AB(herb*) OR AB(decoction*) OR AB(botanic*) | 422 |
| #3 | #1 AND #2 | **42** |

**OASIS**

|  | Searches | Results |
| --- | --- | --- |
| #1 | 치매 AND 한약 | **7** |

**KISS**

|  | Searches | Results |
| --- | --- | --- |
| #1 | 치매 AND 한약 | **6** |

**RISS**

|  | Searches | Results |
| --- | --- | --- |
| #1 | 치매 AND 한약 | **11** |

**KMbase**

|  | Searches | Results |
| --- | --- | --- |
| #1 | 치매 AND 한약 | **7** |

**KCI**

|  | Searches | Results |
| --- | --- | --- |
| #1 | 치매 AND 한약 | **6** |

**CNKI**

|  | Searches | Results |
| --- | --- | --- |
| #1 | (SU='痴呆'+'阿尔茨海默病'+'dementia'+'Alzheimer') AND (SU='精神行为'+'幻觉'+'妄想'+'攻击行为'+'焦虑'+'忧虑'+'烦乱'+'流浪'+'抑郁'+'失眠'+'睡眠'+'不寐'+'BPSD') AND (SU='中药'+'中医药'+'本草'+'汤'+'丸'+'散'+'颗粒'+'胶囊'') | **280** |

**Wanfang data**

|  | Searches | Results |
| --- | --- | --- |
| #1 | (主题:痴呆 or 主题:阿尔茨海默病 or 主题:dementia or 主题:Alzheimer) AND (主题:精神行为 or 主题:幻觉 or 主题:妄想 or 主题:攻击行为 or 主题:焦虑 or 主题:忧虑 or 主题:烦乱 or 主题:流浪 or 主题:抑郁 or 主题:失眠 or 主题:睡眠 or 主题:不寐 or 主题:BPSD) AND (主题:中药 or 主题:中医药 or 主题:本草 or 主题:汤 or 主题:丸 or 主题:散 or 主题:颗粒 or 主题:胶囊) | **960** |
